# Human Condensin I and II Drive Extensive ATP-Dependent Compaction of Nucleosome-Bound DNA

**DOI:** 10.1016/j.molcel.2020.04.026

**Published:** 2020-07-02

**Authors:** Muwen Kong, Erin E. Cutts, Dongqing Pan, Fabienne Beuron, Thangavelu Kaliyappan, Chaoyou Xue, Edward P. Morris, Andrea Musacchio, Alessandro Vannini, Eric C. Greene

**Affiliations:** 1 Department of Biochemistry and Molecular Biophysics, Columbia University Irving Medical Center, New York, NY 10032, USA; 2 Division of Structural Biology, The Institute of Cancer Research, London SW7 3RP, UK; 3 Department of Mechanistic Cell Biology, Max Planck Institute of Molecular Physiology, 44227 Dortmund, Germany; 4 Fondazione Human Technopole, Structural Biology Research Centre, 20157 Milan, Italy

**Keywords:** condensin, SMC complexes, chromosome organization, loop extrusion, single molecule, DNA curtain, electron microscopy, crosslinking mass spectroscopy

## Abstract

Structural maintenance of chromosomes (SMC) complexes are essential for genome organization from bacteria to humans, but their mechanisms of action remain poorly understood. Here, we characterize human SMC complexes condensin I and II and unveil the architecture of the human condensin II complex, revealing two putative DNA-entrapment sites. Using single-molecule imaging, we demonstrate that both condensin I and II exhibit ATP-dependent motor activity and promote extensive and reversible compaction of double-stranded DNA. Nucleosomes are incorporated into DNA loops during compaction without being displaced from the DNA, indicating that condensin complexes can readily act upon nucleosome-bound DNA molecules. These observations shed light on critical processes involved in genome organization in human cells.

## Highlights

• Architecture of ATPγS-bound human condensin II
• Human condensin II possesses two putative DNA binding compartments
• Human condensins drive robust ATP-dependent compaction of nucleosome-bound DNA
• Loop extrusion by human condensins can be symmetric or asymmetric

## Graphical Abstract

**Figure:**
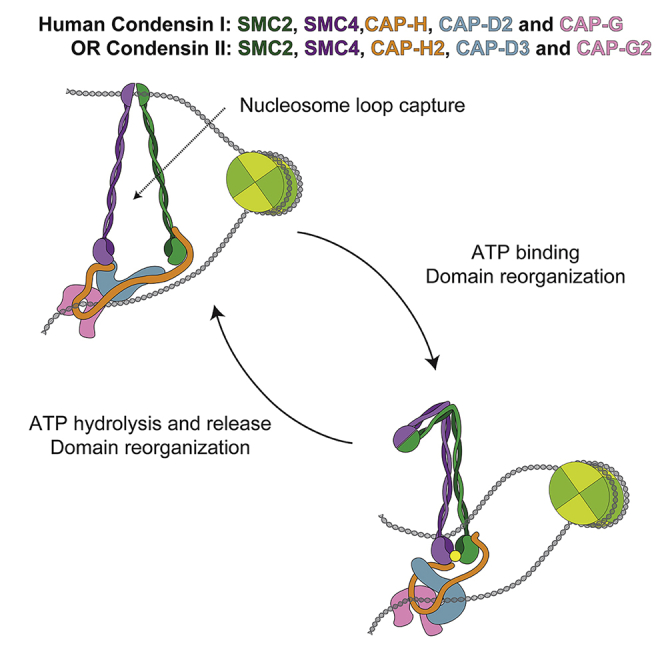

## Teaser

Kong and Cutts et al. present the general architecture of ATPγS-bound human condensin I and II complexes. They demonstrate that both human condensins are ATP-dependent motors that drive robust compaction of nucleosome-bound DNA, in either a symmetric or asymmetric manner, supporting the loop extrusion model under physiological conditions in higher eukaryotes.

## Introduction

SMC (structural maintenance of chromosomes) complexes are essential for chromosome organization (Dekker and Mirny, 2016, Gruber, 2018, Hirano, 2012, Nasmyth, 2001, Nolivos and Sherratt, 2014). Three main SMC families exist in eukaryotes: cohesin; condensin; and the SMC5-6 complex (Hirano, 2006, Jeppsson et al., 2014, Jessberger, 2002, Uhlmann, 2016). Cohesin mediates sister chromosome cohesion and is involved in DNA repair (Losada, 2014, Nasmyth and Haering, 2009). SMC5-6 plays an important role in genome stability (Aragón, 2018). Condensins compact DNA to form mitotic chromosomes and are considered the primary driver of chromosome architecture (Dekker and Mirny, 2016, Hirano, 2016, Mirny et al., 2019).

**Figure 1:**
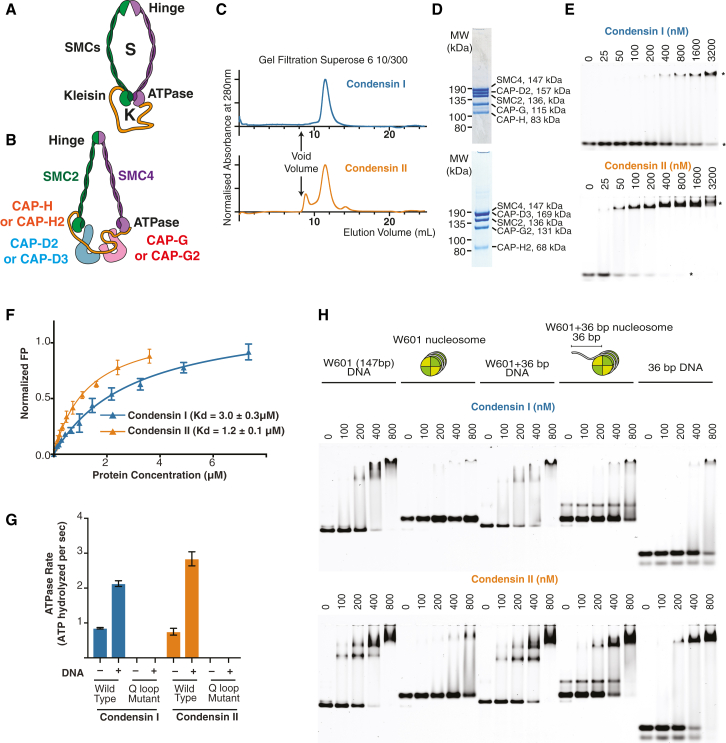
Characterization of Human Condensin I and II (A) SMC complex schematic, showing the SMC (S) and kleisin (K) compartments. (B) Schematic of human condensins. (C) Gel filtration profile of CI and CII. (D) Coomassie-stained SDS-PAGE gel from major peak from gel filtration (MW, molecular weight). (E) DNA binding assays for CI and CII, using a 30-bp Cy5-labeled dsDNA (50 nM); ^∗^ indicates free and bound DNA. (F) Quantification of CI and CII DNA binding affinity by fluorescence polarization (FP) with 30-bp 6FAM-labeled dsDNA. (G) ATP hydrolysis activity of CI and CII ± 100-fold excess of 50-bp dsDNA. (H) CI and CII binding to a 147-bp Widom 601 sequence (W601) or the W601 sequence with the additional 36-bp overhang ± nucleosomes. Error bars indicate one standard deviation.

SMC complexes share a conserved architecture, namely a tripartite ring composed of two SMC proteins and a kleisin family protein (Nasmyth and Haering, 2005, Schleiffer et al., 2003). Each SMC protein has a ∼50-nm-long coiled-coil domain with an ATPase domain formed by the N and C termini at one end and a dimerization “hinge” domain at the other end (Haering et al., 2002, Hirano, 2006). Two SMC proteins dimerize via the hinge domain, bringing the two ATPase half-sites together (Lammens et al., 2004). Recent work on *S. cerevisae* cohesin and *B. subtilis* Smc-ScpA has shown that the ATPase heads can interact in two ways: in the presence of ATP they adopt an “engaged” conformation, referred to as the E state, capable of ATP hydrolysis; upon hydrolysis, ATPase heads interact in a “juxtaposed” orientation, referred to as the J state (Chapard et al., 2019, Vazquez Nunez et al., 2019). The geometry of the SMC complexes yields at least two DNA entrapment compartments: the SMC (S) compartment formed by dimerization of the SMC subunits at the hinge and ATPase domains and the kleisin (K) compartment as a result of kleisin family protein binding to the SMC proteins near each of the ATPase domains (Figure 1A; Chapard et al., 2019, Onn et al., 2007, Sedeño Cacciatore and Rowland, 2019, Vazquez Nunez et al., 2019). Both S and K compartments are thought to exist in either the ATP-bound E state or the ATP-free J state (Chapard et al., 2019, Vazquez Nunez et al., 2019). Condensin and cohesin also have a number of HEAT-repeat proteins, which contribute to DNA binding, ATP hydrolysis, and function (Hassler et al., 2018, Nasmyth and Haering, 2005, Petela et al., 2018). To date, structural studies have focused on individual HEAT subunits or condensin subcomplexes (Chao et al., 2017, Hara et al., 2019, Hassler et al., 2019, Kschonsak et al., 2017, Lee et al., 2016). Although these structures provide invaluable details into specific subunits and isolated interaction surfaces, the general architecture of a full-length SMC complex is still missing.

Lower eukaryotes have a single condensin, but the majority of higher eukaryotes, including humans, have two condensins, condensin I (CI) and II (CII) (Hirano, 2012, Hirano, 2016, Thadani et al., 2012). Although sharing the same SMC subunits (SMC2 and SMC4), condensin I and II have distinct non-SMC regulatory subunits, including the kleisin subunit (CAP-H and CAP-H2, respectively) and a pair of HEAT repeat subunits (CAP-D2/G and CAP-D3/G2, respectively; Figure 1B). Though the non-SMC subunits of condensin I are widely conserved, the sequence of condensin II non-SMC subunits are more divergent, suggesting that these complexes may have distinct activities. Condensin I and II associate with chromosomes at different stages during the cell cycle (Hirota et al., 2004, Ono et al., 2003, Ono et al., 2004). An emerging model suggests that condensin II organizes the prometaphase chromosome into large (200- to 400-kb) outer loops, which are subdivided into smaller (80-kb) inner loops by condensin I (Gibcus et al., 2018, Hirano, 2016, Walther et al., 2018). Thus, the combined actions of both condensins contribute to formation of a nested-loop architecture necessary to achieve the highest level of chromosome compaction.

Although DNA loop formation by SMC complexes has long been anticipated and widely observed *in vivo* (Dekker et al., 2002, Gassler et al., 2017, Gibcus et al., 2018, Nasmyth, 2001, Naumova et al., 2013, Rao et al., 2014, Rao et al., 2017, Schalbetter et al., 2017, Schwarzer et al., 2017, Vian et al., 2018, Wang et al., 2018), the underlying mechanisms remain elusive. Two models describe how condensin might compact DNA: the stochastic crosslinking model, where condensin passively bridges distal chromosomal sites, and loop extrusion, where condensin actively extrudes loops of DNA (Dekker and Mirny, 2016, Nasmyth, 2001, Uhlmann, 2016, van Ruiten and Rowland, 2018). Single-molecule studies have shown that yeast condensin is an ATP-dependent motor protein that can extrude DNA loops, providing support for the loop extrusion model (Ganji et al., 2018, Terakawa et al., 2017). Yeast condensin relies upon asymmetric (one-sided) DNA loop extrusion (Ganji et al., 2018), but theoretical studies have suggested a symmetric (two-sided) loop extrusion mechanism would be necessary for larger mammalian genomes (Banigan and Mirny, 2019).

Structural evidence from yeast cohesin and *B. subtilis* Smc-ScpA suggests that the S compartment in the E state catches DNA loops then passing the DNA into the K compartment on transitioning to the J state (Chapard et al., 2019, Vazquez Nunez et al., 2019). However, loop extrusion *in vivo* is complicated by the fact that eukaryotic genome exists as chromatin (Luger et al., 2012, Rando and Chang, 2009). It is unclear whether human condensins act as DNA motors and how their activities might be affected by the presence of nucleosomes.

To help address these questions, we purified the two human condensin holocomplexes and investigated their properties. We obtained a structural model of condensin II in the engaged (E) state. This model sheds light into SMC holocomplex architecture and suggests that condensin II encompasses two compartments large enough to accommodate double-stranded DNA (dsDNA). We also developed single-molecule assays for observing condensin behavior on nucleosome-bound DNA. We show that human condensins act as ATP-dependent molecular motors that can drive extensive DNA compaction. We observe symmetric (two-sided) loop extrusion, commensurate with theoretical predictions for optimal compaction of the human genome. Furthermore, we show that nucleosomes are readily incorporated into the DNA loops. Remarkably, the nucleosomes do not impede condensin movement and the nucleosomes themselves are neither evicted from the DNA nor displaced from their original binding positions. These findings imply that human condensin can drive genome compaction while stepping over nucleosomes.

## Results

### Purification and Characterization of Human Condensin I and II

Recombinant human CI and CII, each composed of five subunits, were expressed in insect cells. The subunits of these complexes co-eluted in gel filtration, and sample purity was confirmed by SDS-PAGE and mass spectroscopy (Figures 1C and 1D; Table S1). CI and CII could bind DNA and hydrolyze ATP. DNA binding was tested with electrophoretic mobility shift assays (EMSAs) (Figure 1E) and quantified by fluorescence anisotropy (Figure 1F), which indicated that CI has at least a 2-fold lower DNA affinity (K_D_ = 3.0 ± 0.3 μM) compared to CII (K_D_ = 1.2 ± 0.1 μM). CI and CII hydrolyzed ATP at rates of 0.85 ± 0.02 and 0.75 ± 0.10 ATP molecules per second per holocomplex, respectively (Figure 1G). Mutations of the Q-loop (SMC2 Q147L and SMC4 Q229L), which is proposed to coordinate magnesium required for ATP binding, abolished ATPase activity (Figure 1G; Eeftens et al., 2017, Hassler et al., 2019, Hopfner et al., 2000, Hopfner and Tainer, 2003, Löwe et al., 2001, Terakawa et al., 2017). ATP hydrolysis was stimulated 2.5- and 3.8-fold by dsDNA for CI and II, respectively (Figure 1G). These experiments indicate that human CI and CII are both DNA-stimulated ATPases.

In physiological settings, condensins must act on chromatin. However, CI and CII bound very poorly to a 147-bp DNA fragment bound by a nucleosome (Figure 1H). In contrast, CI and CII could bind to a 183-bp substrate bearing a nucleosome (Figure 1H). This 183-bp substrate contains a 36-bp flanking segment that is not bound by nucleosomes, suggesting that, although CI and CII do not efficiently bind to the core nucleosome, they can bind the flanking DNA.

### Analysis of CI and CII by Negative Stain EM

**Figure 2:**
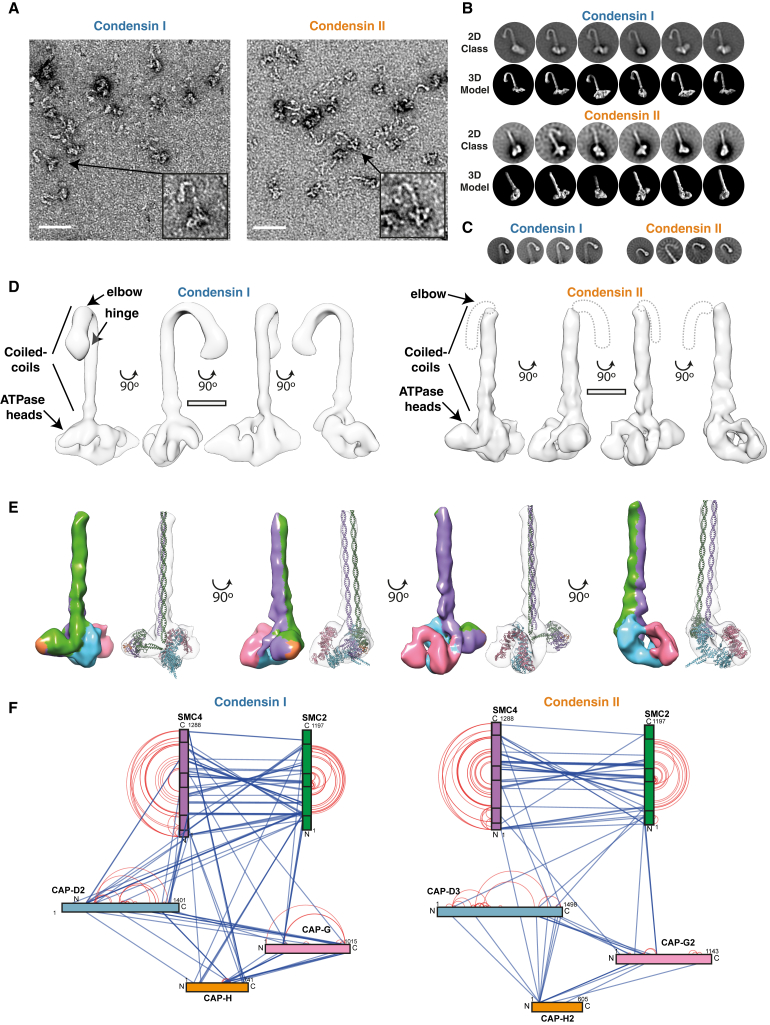
Structural Analysis of CI and CII (A) Negative stain electron micrograph of gradient crosslinked CI and CII; the arrow indicates an example particle (scale bar: 50 nm). (B) 2D classifications of whole CI and CII particles compared to 3D model. (C) 2D classifications, re-centered on the elbow illustrating that this feature is present in the raw data. (D) CI and CII 3D model at 31.8 Å and 20.5 Å (scale bar: 10 nm). (E) Pseudo-atomistic model of CII, with subunits and surface colored as in (F). (F) Network diagram of crosslinks found at least three times in CI and CII in the presence of ATPγS. Intra-subunit crosslinks are shown in red; inter-subunit crosslinks are shown in blue. Boxes within SMC2/4 indicate location of ATPase domains at N and C terminus and central hinge domain. See also Figure S1.

CI and CII were analyzed by negative stain electron microscopy. To trap the ATP-bound E conformational state, CI and CII were pre-incubated with ATPγS and crosslinked using the GraFix method (Kastner et al., 2008). CI and CII samples appeared as ∼60-nm elongated particles (Figure 2A). The hinge, coiled-coil, and HEAT domains were clearly recognizable in micrographs and 2D classifications of both CI and CII (Figure 2B). However, comparison of 2D classes of CI and CII suggested that there may be differences in domain architecture. To better resolve the features in the hinge, a focused 2D classification was performed, highlighting the toroidal shape of the hinge domain and a prominent bend in the coiled coil region ∼15 nm from the hinge, the “elbow.” Bacterial MukBEF and yeast cohesin display similar features (Bürmann et al., 2019, Griese et al., 2010, Kurze et al., 2011, Soh et al., 2015; Figure 2C). 3D electron microscopy (EM) maps of CI and CII were obtained at ∼31.8 Å and 20.5 Å, respectively (Figure 2D). Most particles displayed juxtaposed coiled-coil domains, forming a stem that emerges from the globular domain, which is constituted from the kleisin and HEAT subunits (Figures 2A and 2B), similar to bacterial MukBEF and yeast cohesin (Bürmann et al., 2019, Griese et al., 2010, Kurze et al., 2011, Soh et al., 2015). The maps confirmed similar overall architecture of CI and CII and the conservation of SMC architecture across different species.

### CII Model Suggests Two DNA Compartments

The CII map was of sufficient quality to fit homology models of the SMC2/4 and CAP-D3/G2 and generate a hybrid 3D model (Figure 2E). The structural model of CII suggested the presence of two well-defined cavities (Figure S1A) that were lined with positively charged residues and that the openings were large enough to accommodate dsDNA (Figures S1B–S1D). The first compartment is formed by the engaged ATPase heads and likely corresponds to the E-state S compartment (Chapard et al., 2019). Superimposing the DNA-bound ATPase heads of *Chaetomium thermophilum (Ct)* Rad50 (PDB: 5DAC; Seifert et al., 2016) with the CII-engaged SMC2/4 heads positions the dsDNA within this compartment (Figure S1E). The model of CII includes the C-terminal region of CAP-H2, which binds to the SMC4 ATPase domain, and the putative CAP-H2 path can be traced based on alignment with the CAP-H2 homolog from *Ct* Brn1 (Figures S1E–S1H; Hassler et al., 2019, Kschonsak et al., 2017). The second compartment is formed by the kleisin and HEAT repeat domains and likely corresponds to the E-state K compartment (Chapard et al., 2019). Despite the quality of the EM maps, at this resolution, the pseudo-symmetry of SMC2/4 results in ambiguity of the fitting.

To resolve this ambiguity and to validate the CII model, crosslinking mass spectroscopy was performed in the presence of ATPγS (Figure 2F) using disuccinimidyl dibutyric urea (Pan et al., 2018). The CII model satisfied 83% of crosslinked pairs present. The crosslink from CAP-G2:340 to CAP-D3:1214 was the most abundant non-SMC inter-domain crosslink, which supported the modeled orientation of CAP-G2 and the N-terminal region of CAP-G2 being in close proximity to the C-terminal region of CAP-D3. Analysis of the crosslinked peptides also confirmed engagement of the ATPase heads (crosslinks between SMC2:1079-SMC4:1267 and SMC2:12-SMC4:1187). Multiple crosslinks between the SMC2/4 coiled-coils could be observed, particularly in the region between the elbow and the hinge, suggesting that this region may close independently of the rest of the SMC arms. Interestingly, a cluster of crosslinks between the interface of CAP-G2/D3 with SMC2 and a crosslink between the hinge and coiled-coil region near the ATPase domain violate the distance constrains in the CII model and likely represent an alternate conformation, such as the scrunched conformation observed using atomic force microscopy (AFM) (Ryu et al., 2019). There are also violated crosslinks in the homology model of CAP-D3 around the proboscis, a structural element present in the *Ct* Ycs4, a homolog of CAP-D3, indicating that this feature may not be present in human CAP-D3, in agreement with its poor sequence conservation across species (Hassler et al., 2019).

Our model, together with crosslinking data, suggested that an interaction interface is formed between CAP-D3 and CAP-G2 (Figure S1I). In agreement with this finding, pull-down experiments using insect cell lysates co-expressing CAP-D3 and CAP-G2 indicated that a strep-tagged Cap-D3 could indeed pull down CAP-G2 (Figure S1J), consistent with previous results (Onn et al., 2007). The interaction between these two domains is strengthened by the binding of CAP-H2 to both CAP-D3 and CAP-G2.

Additionally, crosslinking coupled mass spectrometry was performed on CI incubated with ATPγS (Figure 2F). This analysis supported previously reported interactions, such as the interactions between the CAP-H N terminus and SMC2, CAP-H2 residues around 500 with CAP-G, and the CAP-H C terminus with SMC4 (Hara et al., 2019, Onn et al., 2007). Furthermore, crosslinks highlighted the similarities between CI and CII (Figure 2F). Similar enrichment of crosslinking between the coiled-coils of SMC2/4 from the kink elbow to the hinge, between the HEAT domains and the SMC ATPase domains and surrounding coiled-coil regions, and interactions between the hinge and HEAT domains support the similar architecture of CI and CII.

In summary, the crosslinking data validate the proposed general architectural models of CI and CII. Albeit the pseudo-symmetry of SMC2 and SMC4 cannot rule out completely an alternative assignment, the proposed model better fits the observed crosslinks between the coiled-coil domain of SMC2 and CAP-H/H2 N termini, which simultaneously crosslink with the N termini of CAP-D2/D3, for CI and CII, respectively.

### Real-Time DNA Compaction by CI and CII

**Figure 3:**
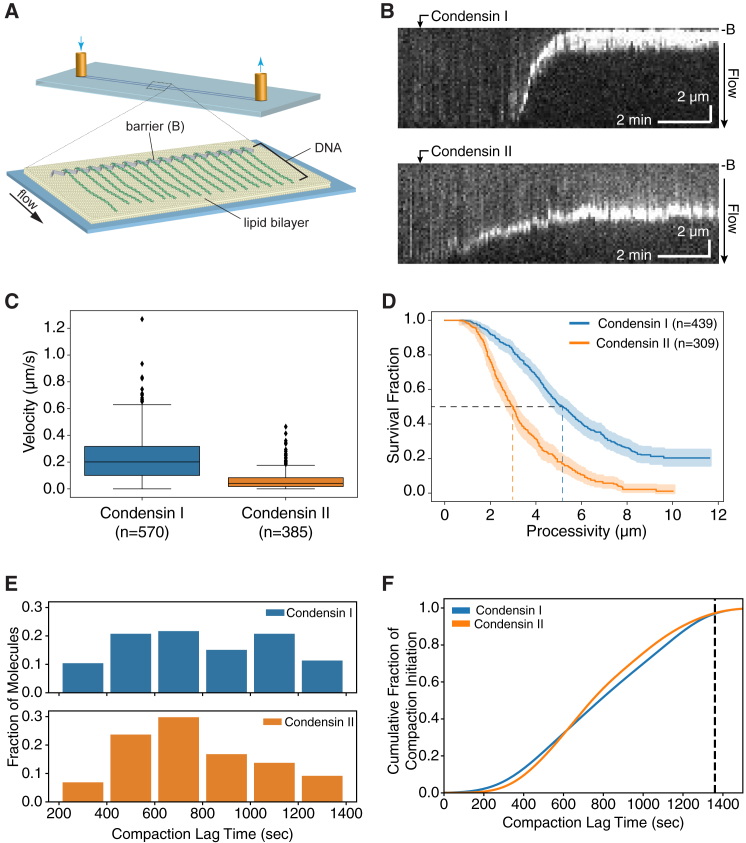
Real-Time DNA Compaction by CI and CII (A) Schematic of the single-tethered DNA curtain assay. (B) Kymographs showing compaction of YOYO1-stained dsDNA molecules by CI (top) and CII (bottom). Arrows indicate the approximate arrival times of condensins in sample chamber. B indicates the position of the Cr barrier. (C) Boxplot of CI and CII compaction velocities on naked DNA. (D) Kaplan-Meier estimated survival functions of compaction processivities on naked DNA. Shaded areas indicate 95% confidence intervals. (E) Distribution of compaction lag times on individual DNA molecules. (F) Cumulative fraction of initiation of DNA compaction events. See also Figures S2 and S3 and Videos S1 and S2.

To visualize DNA compaction, we employed the single-molecule DNA curtain assay using total internal reflection fluorescence microscopy (TIRFM) (Greene et al., 2010). Briefly, YOYO1-stained λ-DNA (48,502 bp) was tethered at one end to the lipid bilayer covering the microfluidic flow cell surface via biotin-streptavidin linkage. The DNA molecules were then aligned at the chromium (Cr) barrier and extended by flow (Figure 3A).

Addition of unlabeled CI (15 nM) or CII (20 nM) in the presence of 4 mM ATP resulted in progressive shortening of >35% of the DNA molecules (CI: n = 110/276; CII: n = 43/117). DNA compaction manifested in a localized increase in the YOYO1 signal that traveled against buffer flow toward the DNA tether point (Figure 3B; Videos S1 and S2). Initially, the localized intensity translocated as a single bright punctum but was later occasionally accompanied by the appearance of an additional signal trailing behind the punctum. Intensity of the trailing signal was higher than that of a single DNA, suggesting the formation of a DNA loop large enough to be extended by hydrodynamic force (Figures S2A, S2C, and S2D). Almost all compaction events were initiated at the free DNA ends (CI: n = 335/345; CII: n = 215/215; Figures S2A–S2D). Rare exceptions of initiations elsewhere along the DNA were only observed in the distal half of the molecule, away from the anchor point and the Cr barrier (Figures S2E and S2F). This preference for compaction to initiate at the free DNA ends is likely due to the fact that the free ends of tethered DNA molecules extended under flow experience much lower force compared to regions closer to the tether points (Doyle et al., 2000, Larson et al., 1997, Perkins et al., 1995). The enhanced flexibility near the free DNA ends would allow for transient loop formation and capture by condensin and facilitate the initiation of productive DNA compaction.

Video S1. Example of Single-Tethered DNA Compaction by Human Condensin I, Related to Figure 3 This video shows a YOYO1-stained single-tethered DNA molecule being fully compacted, from bottom to top, by unlabeled human condensin I (*left*); alongside the kymograph of the compaction (*right*). Compaction was completed at ∼300 s mark. Vertical and horizontal scale bars represent 2 μm and 60 s, respectively.

Video S2. Example of Single-Tethered DNA Compaction by Human Condensin II, Related to Figure 3 This video shows a YOYO1-stained single-tethered DNA molecule being fully compacted, from bottom to top, by unlabeled human condensin II (*left*); alongside the kymograph of the compaction (*right*). Compaction was completed at ∼415 s mark. Vertical and horizontal scale bars represent 2 μm and 60 s, respectively.

### DNA Compaction Requires ATP Hydrolysis by CI and CII

We conducted controls in the presence of the non-hydrolyzable ATP analog ATPγS. These experiments showed no evidence for CI- or CII-mediated DNA compaction in the presence of 4 mM ATPγS (Figures S2G and S2H). However, when ATPγS was replaced with 4 mM ATP, the same DNA molecules were readily compacted (Figures S2G and S2H). As an additional control, we tested the CI and CII ATP hydrolysis-deficient Q-loop mutants (SMC2 Q147L and SMC4 Q229L; Hassler et al., 2019, Hopfner et al., 2000, Hopfner and Tainer, 2003, Löwe et al., 2001). For brevity, we refer to these mutants as CIQ and CIIQ, respectively. In the presence of 4 mM ATP, CIQ failed to compact any of the DNA (Figure S2I). We also observed no DNA compaction with CIIQ, but note that, at later time points, this ATPase-deficient mutant led to increased nonspecific sticking of DNA to the flow cell surface, so evaluation of these data were restricted to the first ∼5 min of the measurements (Figure S2J). Together, these experiments demonstrate that DNA compaction was dependent upon condensin ATP hydrolysis activity.

### Quantification of DNA Compaction Characteristics

Quantification of DNA compaction yielded velocities 0.201 (0.216) μm/s (median and interquartile range [IQR], same below; or ∼908 bp/s; see STAR Methods) and 0.040 (0.066) μm/s (or ∼182 bp/s) for CI (n = 570) and CII (n = 385), respectively (Figure 3C). In addition to higher compaction velocities, CI also exhibited higher processivity relative to CII, compacting ∼5.2 μm (∼23.5 kbp; n = 439) of DNA, compared to ∼3.0 μm (∼13.6 kbp; n = 309) by CII (Figure 3D). These findings suggest that CI can compact longer tracts of DNA much more rapidly than CII.

Analysis of compaction lag times revealed that the initiation of individual DNA compaction events was highly stochastic (Figure S3). Lag times were distributed over the entire ∼20-min observation period (Figures 3E and 3F). This observation is consistent with the low K_D_ values measured in bulk biochemical assays, suggesting lack of cooperativity in the initiation of DNA compaction.

Remarkably, the DNA molecules undergoing active compaction were often (CI: ∼31.5%; CII: ∼11.7%) suddenly released, in a single step within one frame (5 s) of data collection (Figures S2A, S2B, and S2D). These instantaneous events are distinct from gradual reversals, where YOYO signal puncta of compacting DNA molecules travel away from the barrier over several time frames. This type of sudden release was most consistent with the spontaneous dissociation of condensin from the DNA or the sudden rupture of a condensin-DNA contact leading to complete disruption of the compacted DNA loops, allowing the molecule to return to its fully extended length. An important conclusion from these observations is that the DNA compaction events were completely reversible.

### CI and CII Track with the Compacting Loops of DNA

**Figure 4:**
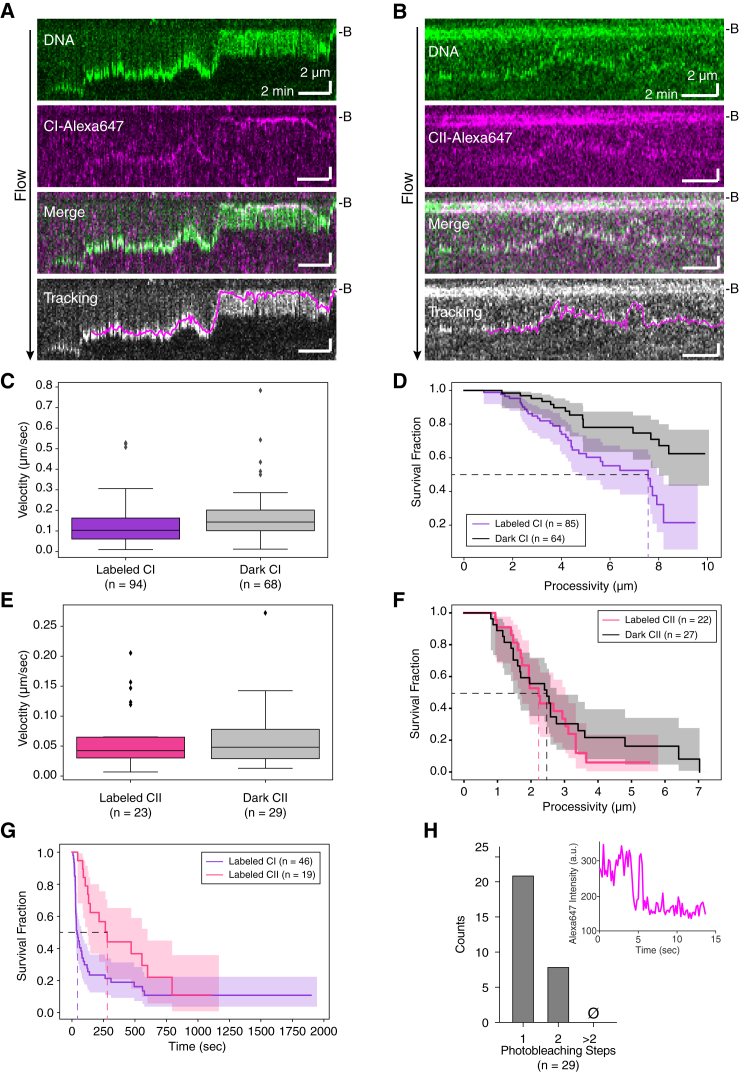
Visualization of Fluorescently Labeled Human Condensin during DNA Compaction (A and B) Kymographs showing compaction of a YOYO1-stained dsDNA by Alexa-Fluor-647-labeled human CI (A) and CII (B), respectively. Bottom panels show overlays of tracked trajectories of labeled condensins (magenta) with DNA signal. (C and D) Compaction velocities (C) (labeled CI median [IQR]: 0.103 [0.102] μm/s or ∼465 bp/s; dark CI median [IQR]: 0.143 [0.100] μm/s or ∼645 bp/s) and processivities (D) (labeled CI half-life: 7.56 μm or ∼34.1 bp; dark CI half-life: not determined), respectively, of YOYO-1 signal puncta co-localized and tracking with labeled or dark CI-Alexa Fluor 647. (E and F) Compaction velocities (E) (labeled CII median [IQR]: 0.042 [0.035] μm/s or ∼189 bp/s; dark CII median [IQR]: 0.048 [0.049] μm/s or ∼217 bp/s) and processivities (F) (labeled CII half-life: 2.22 μm or ∼10.0 kbp; dark CII half-life: 2.47 μm or ∼11.1 kbp), respectively, of YOYO-1 signal puncta co-localized and tracking with labeled or dark CII-Alexa Fluor 647. (G) Survival curves of DNA binding times of Alexa-Fluor-647-labeled CI and CII in single-tethered DNA compaction assays (CI half-life: 46 s; CII half-life: 280 s). (H) Histogram of the number of photobleaching steps for CI-Alexa Fluor 647 complexes that were involved in DNA compaction. Inset: representative one-step photobleaching trace of CI-Alexa Fluor 647 is shown. See also Figure S4 and Videos S3 and S4.

Our data were consistent with a model in which CI and CII co-localized with the compacting ends of the DNA. To directly test the prediction that condensin tracked with the compacting DNA loops, we conjugated Alexa Fluor 647 fluorophore to purified ybbR-tagged CI and CII, achieving ∼70% overall labeling efficiency (Figure S4; Yin et al., 2006). In agreement with our hypothesis, fluorescently labeled CI and CII were observed to co-localize and track with the DNA puncta that formed during compaction, indicating that CI and CII were directly localized within the compacting DNA loops (Figures 4A and 4B; Videos S3 and S4).

Video S3. Example of Single-Tethered DNA Compaction by Alexa-Fluor-647-Labeled Human Condensin I, Related to Figure 4 This video shows a YOYO1-stained single-tethered DNA molecule (green) being compacted, from bottom to top, by Alexa647-labeled human condensin I (magenta); alongside the kymograph (*right*) of the compaction from the DNA channel (green), superimposed with tracked trajectory of the labeled CI (*magenta*). Vertical and horizontal scale bars represent 2 μm and 60 s, respectively.

Video S4. Example of Single-Tethered DNA Compaction by Alexa-Fluor-647-Labeled Human Condensin II, Related to Figure 4 This video shows a YOYO1-stained single-tethered DNA molecule (green) being compacted, from bottom to top, by Alexa647-labeled human condensin II (magenta); alongside the kymograph (*right*) of the compaction from the DNA channel (green), superimposed with tracked trajectory of the labeled CI (*magenta*). Vertical and horizontal scale bars represent 2 μm and 60 s, respectively.

### Single Condensins Drive DNA Compaction

Experiments with Alexa-Fluor-647-labeled condensins contained populations of compacting DNA molecules associated with either labeled (CI: ∼53%; CII: ∼42%) or dark proteins (CI: ∼47%; CII: ∼58%). We attribute the dark population to proteins that were either unlabeled or photobleached. For both CI and CII, compaction velocities were similar whether or not the protein was labeled (Figures 4C and 4E for CI and CII, respectively). Processivities of labeled and dark CII were also comparable (Figure 4F), whereas that of dark CI was not determined due to over 50% of the DNA compaction events reaching the Cr barrier, resulting in a processivity of ≥48 kb (Figure 4D). We note that the median velocity of labeled CI was reduced by ∼50% relative to unlabeled CI (p < 0.001; Mann-Whitney *U* test). However, the median CII compaction velocity, as well as processivities of both CI and CII, was comparable across experiments with labeled and unlabeled condensins. Notably, the observation of DNA molecules undergoing active compaction but not being associated with a fluorescent condensin in reactions with a mixed population of labeled and unlabeled proteins suggested that only a small number of condensin complexes were associated with each DNA. Lastly, we were also able to measure DNA binding times of labeled CI and CII (Figure 4G), where the half-life of CII was approximately six times longer than that of CI (p = 0.037; log rank test), qualitatively recapitulating previous observations of CI being more dynamic in binding than CII (Gerlich et al., 2006).

We performed photobleaching experiments to assess the number of condensin complexes bound to each DNA. Remarkably, in ∼70% of the compaction events (n = 21/29), we observed single-step photobleaching events, suggesting a single CI-Alexa Fluor 647 was compacting the DNA molecule (Figure 4H). The remaining DNA-bound CI-Alexa Fluor 647 complexes (n = 8/29) exhibited two photobleaching steps. Events involving more than two steps were not observed. These results suggest that just one or perhaps two condensin complexes were sufficient to drive the compaction of tens of thousands of kilobases of DNA.

### Visualizing Loop Extrusion by CI and CII

**Figure 5:**
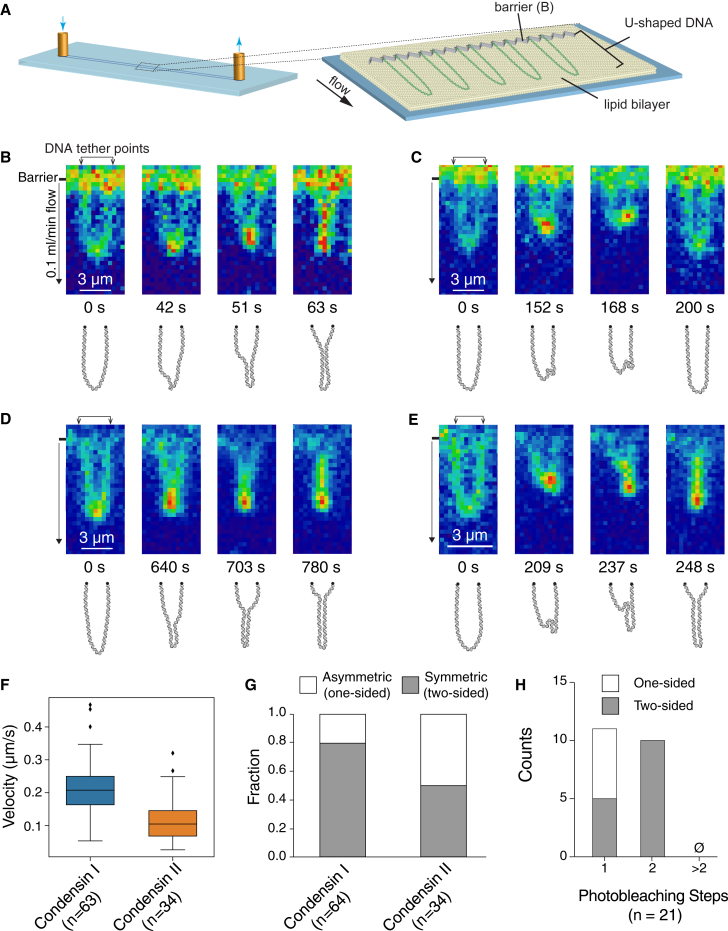
Loop Extrusion of U-Shaped DNA by CI and CII (A) Schematic of U-shaped DNA curtain assay. (B and C) Snapshots and schematics of loop extrusion of YOYO1-stained U-shaped DNA molecules by CI in symmetric (two-sided; B) or asymmetric (one-sided; C) manner. (D and E) Snapshots and schematics of loop extrusion of YOYO1-stained U-shaped DNA by CII in symmetric (two-sided; D) or asymmetric (one-sided; E) manner. (F) Boxplot of loop extrusion velocities. (G) Bar graph comparing the number of asymmetric (one-sided) and symmetric (two-sided) compaction events. (H) Histogram of the number of photobleaching steps for Alexa Fluor 647-CI with one-sided or two-sided events. See also Figure S5 and Videos S5 and S6.

Loop extrusion has emerged as a widely accepted model for DNA compaction by condensins (Alipour and Marko, 2012, Fudenberg et al., 2017, Goloborodko et al., 2016a, Goloborodko et al., 2016b, Hassler et al., 2018). We sought to visualize DNA looping using YOYO1-stained DNA molecules that were biotinylated on both ends and tethered to the lipid bilayer in a U-shaped configuration (Figure 5A), similar to previous loop extrusion assays for yeast condensin (Ganji et al., 2018). For both CI and CII, puncta were observed first forming at the distal end of the U-shaped DNA and then moved progressively toward the tether points, resulting in Y-shaped DNA (Figures 5B–5E). Importantly, the U-shaped DNA remained unchanged in controls with wild-type protein with 4 mM ATPγS or Q-loop mutants in the presence of 4 mM ATP (Figures S5A–S5D). We quantified the progression of DNA puncta as a measure of loop extrusion rates, obtaining median velocities for CI and CII of 0.208 (0.087) μm/s (∼1,056 bp/s) and 0.105 (0.077) μm/s (∼535 bp/s), respectively (Figure 5F). These rates were comparable to the single-tethered DNA compaction assays and are in the same range as those reported for yeast condensin, which varied between 200 bp/s and 1,000 bp/s (Ganji et al., 2018).

The manner in which the U-shaped DNA molecules were acted upon by CI and CII could be categorized as either symmetric (two-sided; Figures 5B, 5D, and S5E; Video S5) or asymmetric (one-sided; Figures 5C, 5E, and S5E; Video S6). We first classified events according to the symmetry in which the DNA puncta were progressively compacted and found that 80% of all events involving CI were symmetric (two-sided) although the remaining 20% were asymmetric (one-sided; Figure 5G). In contrast, assays with CII yielded approximately equal populations of symmetric (two-sided) and asymmetric (one-sided) DNA compaction events (Figure 5G). These observations contrast with yeast condensin, which exhibit 100% asymmetry (Ganji et al., 2018).

Video S5. Symmetric U-Shaped DNA Compaction by Human Condensin I, Related to Figure 5 This video shows a YOYO1-stained U-shaped DNA molecule being compacted symmetrically compacted by unlabeled human condensin I. Scale bar: 1 μm.

Video S6. Asymmetric U-Shaped DNA Compaction by Human Condensin II, Related to Figure 5 This video shows a YOYO1-stained single-tethered DNA molecule being asymmetrically compacted by unlabeled human condensin II. Scale bar: 1 μm.

In photobleaching experiments of CI-Alexa Fluor 647 in U-shaped DNA compaction assays, both one-step and two-step events were observed, indicating either one or two condensins acting on the DNA, respectively (Figure 5H). However, all of the two-step photobleaching events exhibited symmetric (two-sided) behavior, whereas 45% of the one-step events were symmetric (two-sided) with the rest (55%) being asymmetric (one-sided; Figure 5H).

### CI and CII Can Move Loops of DNA

**Figure 6:**
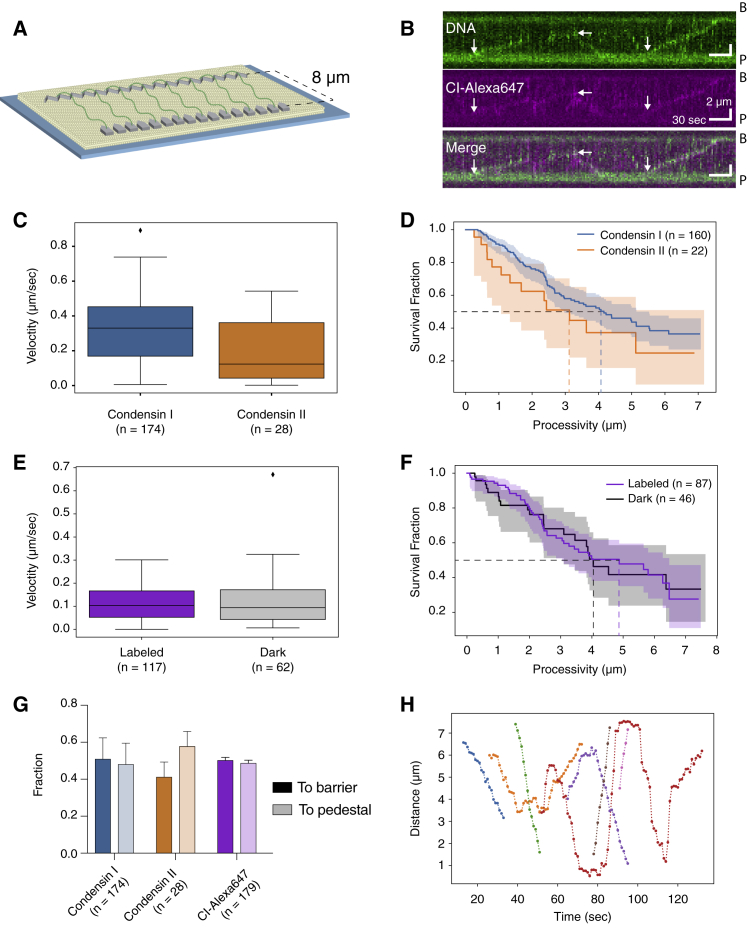
Translocation of CI and CII on Double-Tethered DNA (A) Schematic of double-tethered DNA curtain assay. (B) Representative kymographs of Alexa Fluor 647-CI translocating on double-tethered λ-DNA. White arrows indicate starting points of individual translocation events. (B and P indicate the positions of Cr barrier and pedestal, respectively.) (C and D) Translocation velocities (C) (CI median [IQR]: 0.33 [0.28] μm/s or ∼2,002 bp/s; CII median [IQR]: 0.12 [0.32] μm/s or ∼746 bp/s) and processivities (D) (CI half-life: 4.16 μm or ∼25.2 kbp; CII half-life: 3.13 μm or ∼19.0 kbp), respectively, of YOYO-1 signal puncta along double-tethered DNA in the presence of unlabeled CI or CII. (E and F) Translocation velocities (E) (labeled CI median [IQR]: 0.103 [0.114] μm/s or ∼624 bp/s; dark CI median: 0.094 [0.129] μm/s or ∼570 bp/s) and processivities (F) (labeled CI half-life: 4.87 μm or ∼29.5 kbp; dark CI half-life: 4.04 μm or ∼24.5 kbp), respectively, of YOYO-1 signal puncta along double-tethered DNA molecules co-localized and tracking with labeled or dark CI-Alexa Fluor 647. (G) Fractions of unlabeled or labeled condensin translocation events toward the barrier (dark colors) or the pedestal (light colors). Data are represented as mean ± SD. (H) Representative single-particle tracking trajectories of unlabeled CI translocation.

Yeast condensin is a mechanochemical motor, capable of ATP hydrolysis-dependent translocation on DNA (Terakawa et al., 2017). In these assays, translocation was decoupled from loop extrusion and compaction because the DNA was anchored in an extended state (Greene et al., 2010). Here, we sought to establish whether this linear translocation activity is conserved in human proteins. In contrast to yeast condensin, we observed that neither human CI nor CII translocated on λ-DNA molecules double tethered at 12 μm length, corresponding to a mean DNA fractional extension $\left(X/L_{0}\right)$ of 0.75 (not shown). However, when the tether length was reduced to 8 μm (Figure 6A), both CI and CII exhibited extensive translocation activity, which manifested as localized high-intensity YOYO1 signal puncta traversing the DNA (Figure 6B, top). These movements proceeded over extended periods of time in a unidirectional manner, although occasional reversals were also observed (Figure 6H). Such behavior was not observed in the absence of condensins or in assays with either 4 mM ATPγS or with the Q-loop mutants (not shown). Moreover, the intensity of the YOYO1 signal puncta did not increase or otherwise change appreciably as they moved along the DNA in either direction (Figure 6B, top). Finally, experiments using Alexa-Fluor-647-labeled CI confirmed that CI co-localized with the YOYO1 puncta and tracked with these puncta (Figure 6B).

Single-particle tracking analysis revealed a median velocity of CI almost three times that of CII (Figures 6C and 6D). Similar to results from single-tethered DNA compaction experiments with CI-Alexa Fluor 647, labeling of CI reduced its median translocation velocity by approximately 3-fold (p < 0.001; Mann-Whitney *U* test), with negligible difference between the labeled and dark fractions (Figure 6E). Processivities of labeled and dark CI also remained comparable to the unlabeled protein (Figure 6F). These observations suggest that CI and CII can stimulate DNA loop formation and move the base of these loops along the length of the DNA (Figure 6B).

### Compaction of Nucleosome-Bound DNA

**Figure 7:**
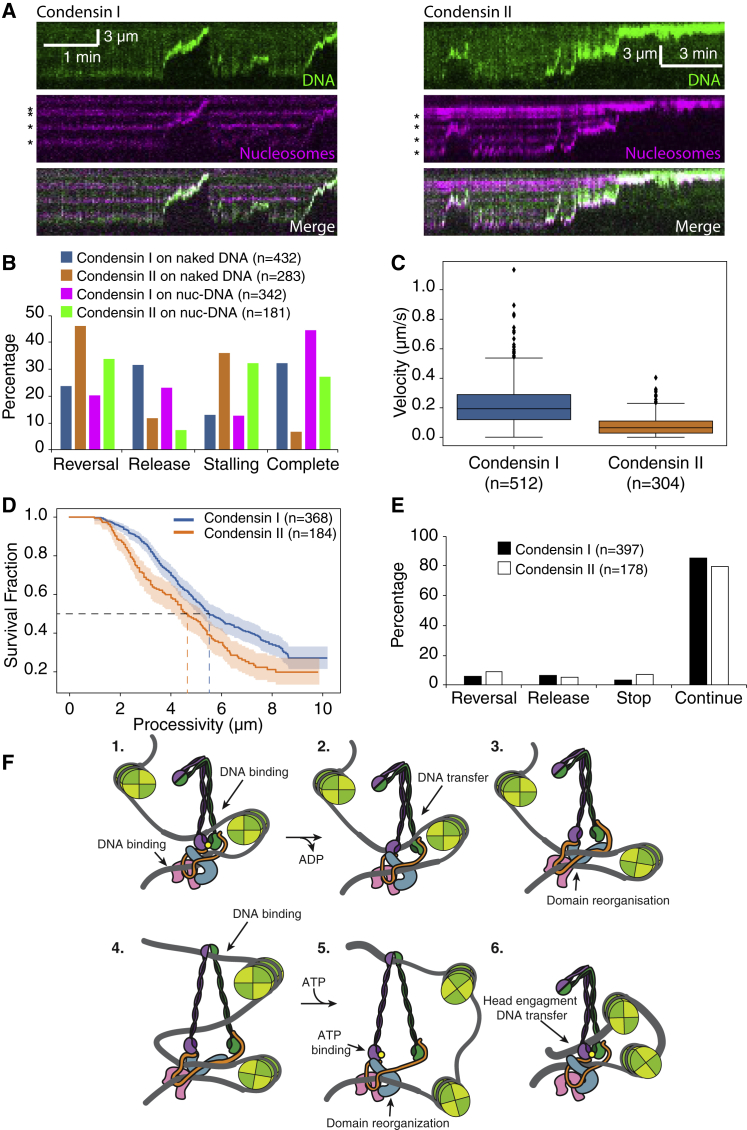
Compaction of Nucleosome-Bound DNA (A) Kymographs showing CI (left)- and CII (right)-mediated compaction of single-tethered, YOYO1-stained DNA (green) bound by ATTO647-labeled nucleosomes (magenta). (B) Percentages of event types that resulted in termination of DNA compaction events by condensin on either naked DNA or nucleosome-bound DNA (nuc-DNA). (C) Boxplot of compaction velocities of CI and CII on nucleosome-bound DNA. (D) Kaplan-Meier estimated survival functions of compaction processivities on nucleosome-bound DNA. Shaded areas indicate 95% confidence intervals. (E) Outcomes of collisions between condensins and individual nucleosomes. (F) Model for DNA compaction by human condensins. See also Figure S6 and Videos S7 and S8.

Loop extrusion is a prevailing model for genome organization by condensin (Alipour and Marko, 2012, Gibcus et al., 2018, Goloborodko et al., 2016a, Goloborodko et al., 2016b, Nasmyth, 2001, Takahashi and Hirota, 2019). For loop extrusion to occur in physiological settings, condensin must be capable of acting upon DNA that is bound by nucleosomes or nucleosomes must be removed from DNA (Dekker and Mirny, 2016, Khorasanizadeh, 2004, Luger et al., 2012, Mirny et al., 2019, Robellet et al., 2017). To help establish whether condensins could act on nucleosome-bound DNA, we investigated how CI and CII responded to the presence of single nucleosomes. To this end, we assembled ATTO-647N-labeled recombinant *X. laevis* histone octamers on λ-DNA under conditions that yielded ∼3 to 4 nucleosomes per DNA (Figure S6; Dyer et al., 2004, Luger et al., 1999, Xue et al., 2019). We observed extensive compaction of nucleosome-bound DNA by both CI and CII (Figure 7A; Videos S7 and S8). The nucleosomes were readily incorporated into the compacted DNA, as was evident by the co-localization of ATTO-647 and YOYO1 signal as the compacted punctum traveled toward the barrier (Figure 7A). Most importantly, when the compaction events underwent spontaneous reversal, the nucleosomes remained bound at their original locations prior to the onset of compaction (Figure 7A).

Video S7. Nucleosome DNA Compaction by Human Condensin I, Related to Figure 7 This video shows a YOYO1-stained, nucleosome (magenta)-bound, single-tethered DNA molecule (green) being compacted, from bottom to top, by unlabeled human condensin I; alongside the kymograph of the compaction. Nucleosome-bound DNA was initially fully compacted by ∼170 s mark, before released and compacted again. Vertical and horizontal scale bars represent 2 μm and 60 s, respectively.

Video S8. Nucleosome DNA Compaction by Human Condensin II, Related to Figure 7 This video shows a YOYO1-stained, nucleosome (magenta)-bound, single-tethered DNA molecule (green) being compacted, from bottom to top, by unlabeled human condensin II; alongside the kymograph of the compaction. Nucleosome-bound DNA was initially partially compacted by ∼80 s mark, before released and fully compacted by ∼570 s mark. Vertical and horizontal scale bars represent 2 μm and 60 s, respectively.

Next, we sought to clarify whether behavior of condensins was significantly altered by the presence of nucleosomes. For this analysis, we classified the termination of processive compaction events into four categories: (1) reversal, where condensin undergoes a gradual reversal in direction; (2) release, where the compacted DNA is released in a single step event; (3) stalling, where compaction halts; or (4) complete compaction that goes all the way to the Cr barrier before stopping. The general behavior of human CI and CII was comparable on naked DNA and nucleosome-bound DNA (Figure 7B). Moreover, quantitation of nucleosome-bound DNA compaction confirmed that the nucleosomes did not significantly affect compaction velocity or processivity. Median velocities of CI and CII on nucleosome-bound DNA were 0.194 (0.169) μm/s or ∼1,032 bp/s and 0.065 (0.082) μm/s or ∼345 bp/s, respectively (Figure 7C). Median processivities for CI and CII on nucleosome-bound DNA were 5.52 μm (or ∼29.4 kbp) and 4.63 μm (or ∼24.6 kbp), respectively (Figure 7D). We conclude that nucleosomes did not substantially impact the general properties of CI or CII but interestingly increased both the velocity and processivity of CII by ∼50%.

Notably, when condensin encountered individual nucleosomes, approximately 80% of all compaction events proceeded completely unimpeded without stalling, releasing, or reversing direction (CI: n = 336/397; CII: n = 141/178; Figure 7E). Most (∼90%) of these encounters did not exhibit any pausing within our temporal resolution (5 s; CI: n = 304/336; CII: n = 122/141), indicating that the nucleosomes did not hinder either CI or CII movement. Together, our findings suggest that human CI and CII can bypass nucleosomes while actively compacting DNA.

## Discussion

To better understand the molecular mechanisms of eukaryotic condensins, we have characterized the properties of human CI and CII. Our results have important implications for understanding how condensin complexes act while controlling the spatial organization of the genome in physiological settings.

### Eukaryotic Condensins Act as Molecular Motors

Classical models suggested that DNA loop extrusion could serve as an important mechanism for driving the spatial organization of chromosomes (Kimura et al., 1999, Nasmyth, 2001, Riggs, 1990). These models for loop extrusion as a primary means of chromosome organization have received extensive support from detailed theoretical studies (Fudenberg et al., 2016, Goloborodko et al., 2016a, Goloborodko et al., 2016b, Sanborn et al., 2015), as well as a range of *in vivo* studies (Ganji et al., 2018, Gibcus et al., 2018, Rao et al., 2014, Rao et al., 2017, Schwarzer et al., 2017, Vian et al., 2018).

Experimental support for loop extrusion as a means of chromosome spatial organization also comes from studies of *B. subtilis* and *C. crescentus*, revealing time-dependent changes in chromosome arm contacts driven by SMC complexes (Tran et al., 2017, Wang et al., 2017, Wang et al., 2018). In addition, single-molecule studies have shown that the yeast condensin can act as a molecule motor and promote loop extrusion (Ganji et al., 2018, Terakawa et al., 2017). Our work extends these observations to the human condensins, providing a crucial demonstration that the motor activity of SMC complexes is widely conserved. Moreover, the range of values we report for loop extrusion by human condensins (∼1,000 bp/s and ∼500 bp/s for CI and CII, respectively) is comparable to the *in vitro* loop extrusion rate for the yeast condensin complex (≤1,500 bp/s; Ganji et al., 2018) and the *in vivo* rate of loop formation for the *B. subtilis* SMC proteins (∼830 bp/s; Wang et al., 2018). In addition, we observed spontaneous disruptions of compacted DNA loops in all of our single-molecule assays. Dissolution of DNA loops during compaction has been shown to be an important feature of chromosome compaction and segregation (Goloborodko et al., 2016a).

In striking contrast to yeast condensin (Ganji et al., 2018), we find that a large proportion of loop extrusion events (∼50%–80%) observed for both human condensins occur through a two-sided symmetric mechanism that was especially prominent for human CI (∼80% symmetric). Notably, recent theoretical studies predicted that a symmetric two-sided extrusion mechanism would be necessary to accommodate the compaction of mammalian chromosomes (Banigan and Mirny, 2019). The two-sided symmetry may be a result of two asymmetric condensin complexes, as evidenced by our observation that two-step photobleaching events of CI-Alexa Fluor 647 were also exclusively two sided. More importantly, strictly symmetric loop extrusion has been recently reported for human cohesin, suggesting that this mechanism may be common to human SMC complexes (Davidson et al., 2019, Kim et al., 2019).

### Human Condensin Can Function on Nucleosome-Bound DNA

For loop extrusion to serve as a model for chromosome organization, it is essential that condensin be able to interact with nucleosome-bound DNA. In principle, one can envision three potential scenarios: condensin may be able to remodel or remove nucleosomes from DNA through its inherent ATP-dependent motor activity; auxiliary nucleosome remodeling factors may help condensin to act upon nucleosome-bound DNA; or condensin may have an inherent ability to act upon nucleosome-bound DNA with no need for nucleosome removal or remodeling. Our results support that later scenario. Remarkably, the nucleosomes themselves are readily incorporated into the DNA loops and the apparent positions of the nucleosomes remain unaltered. This finding implies that human condensins may act upon chromatin fibers in crowded physiological settings and could do so without altering any epigenetic information that may be contained within the underlying nucleosomes. Combined with the recent report that human cohesin can also actively compact nucleosome-bound DNA (Kim et al., 2019), our results suggest a conserved mechanism for how human SMC proteins function on chromatin.

### Architecture and Proposed Mechanism of Condensins

Here, we present the first structural model of a full condensin holocomplex. As ATP hydrolysis, DNA binding, and loop extrusion of CI and CII are all conserved from yeast, the structural rearrangements and mechanism of these is likely to be comparable; hence, the model presented enables existing structures of related condensin subunits to be fitted and analyzed in the context of the whole complex.

The crystal structure of the engaged ATPase heads from SMC-like protein Rad50 (PDB: 5DAC) has shown that DNA can bind to a positively charged grove formed by head dimerization (Seifert et al., 2016). Many of these positively charged residues are present in other SMC proteins (Hassler et al., 2018), and mutation of these residues in Smc-ScpAB resulted in a severe growth phenotype, suggesting they are essential for function (Vazquez Nunez et al., 2019). Alignment of the Rad50 DNA-bound structure with our CII model suggests this DNA binding site is conserved and is likely responsible for the DNA entrapment mediated by the S compartment (Figure S1E; Chapard et al., 2019). Superimposition of the NMR structure of a fusion protein made of the N terminus of Brn1 and two helices of SMC2 (Hassler et al., 2019) with the CII model suggests that the SMC2 helices in this region are highly flexible. Structural data and sequence analysis indicate a break in the coiled-coil propensity of this region in several SMC proteins, referred to as the “joint” (Diebold-Durand et al., 2017, Gligoris et al., 2014), in agreement with the sharp bend in the coiled-coils observed in the presented EM model near the ATPase domains (Figure S1F).

The structure of the yeast CAP-G2 homolog Ycg1 has been determined bound to Brn1 and DNA (Kschonsak et al., 2017). Overlay of this structure with our model suggests that, if DNA binds CAP-G2 in a similar manner, it would be on the outward face of the complex (Figure S1G). This orientation is supported by abundant crosslinks between CAP-G2 and CAP-D3 (CAP-G2:340 to CAP-D3:1214). Thus, taking in consideration this additional DNA binding site on CAP-G2, three distinct DNA binding regions could coexist in the closed conformation of CII.

The CII model presented here is of the engaged form, with ATP heads together and CAP-D3 contacting CAP-G2. Recent work of *Ct* condensin indicates that, in the absence of ATP, the CAP-D3 homolog Ycs4 binds to the SMC4 ATPase head (Figure S1H; Hassler et al., 2019). In our model, the homologous surface of CAP-D3 that binds SMC4 instead interacts with CAP-G2, suggesting the hypothesis that CAP-D3 could switch between binding SMC4 in the absence of ATP and to binding CAP-G2 in the presence of ATP (Figure S1H). This switch would require a large conformational change involving opening of the ATPase heads and movement of the SMC4 ATPase head domain, CAP-D3, and CAP-G2. Such a conformational change could enable passage between the DNA binding compartment S made by SMC2/4 into the K compartment created by the kleisins and HEAT repeat proteins, as previously proposed for the mechanism of SMC complexes (Vazquez Nunez et al., 2019).

The crosslinking data and structural model suggest domain architecture could also be similar to cohesin. Previous crosslinking studies of cohesin suggest the N terminus of Pds5B interacts with the coiled-coil region of SMC3 near the binding site of the N terminus of Scc1 to SMC3, and the C terminus of Pds5B bind SA1 (Hons et al., 2016, Huis in ’t Veld et al., 2014), where Pds5B, SMC3, and SA1 interact with the cohesin kleisin, Scc1, in similar positions to CAP-D3, SMC2, and CAP-G2 interaction with the CII kleisin, CAP-H2, respectively (Chan et al., 2013, Gligoris et al., 2014, Roig et al., 2014).

We propose a possible model of loop extrusion (Figure 7F). We speculate that condensin may translocate on DNA using some type of stepping motion in which conformational changes in the arm domains are coupled to changes in the ATP-bound state of the ATPase head domains (Terakawa et al., 2017). In step 1, two DNA entrapment compartments are available in the ATP-bound form, in addition to a potential DNA binding site on CAP-G2. In step 2, ATP hydrolysis induces separation of the ATPase heads, opening the S compartment and enabling DNA transfer. In step 3, in absence of ATP, CAP-D3 binds to SMC4, trapping the newly transferred strand. In step 4, disruption of head dimerization enables SMC arms to open, revealing new DNA binding sites in SMC arms and hinge. As condensin does not readily act on DNA under tension, relaxed DNA loops are likely the preferred substrate. Separation of the SMC arms could result in the formation of an opening as large as ∼30 nm across, enabling passage of ∼11-nm nucleosome (Luger et al., 1997). In step 5, ATP binding to SMC4 ATPase displaces CAP-D3, enabling loop fusion and promoting ATPase head dimerization. In step 6, ATPase head dimerization promotes SMC arm dimerization, resulting in loss of DNA binding to the hinge and/or arms in favor of binding to dimerized ATPase heads. Extension of this mechanism to a scenario in which two condensins act concurrently in opposing directions would readily accommodate two-sided symmetrical DNA loop extrusion.

## STAR★Methods

### Key Resources Table

**Table:** 

REAGENT or RESOURCE	SOURCE	IDENTIFIER
**Antibodies**		

Mouse monoclonal hCAP-D3 antibody	Santa Cruz	Cat# Sc-81597; RRID: AB_2235818
Rabbit polyclonal hCAP-G2 antibody	Bethyl	Cat# A300-605A; RRID: AB_2149995
Anti-mouse IgG (H+L) (DyLight 800 4X PEG Conjugate)	Cell Signaling	Cat# 5257; RRID: AB_10693543
Anti-rabbit IgG (H+L) (DyLight 800 4X PEG Conjugate)	Cell Signaling	Cat# 5151; RRID: AB_10697505
Anti-Digoxigenin, Fab fragments	Roche	Cat# 11214667001; RRID: AB_514494

**Bacterial and Virus Strains**		

DH10EMBacY competent cells	Geneva Biotech	N/A
BL21(DE3) pLysS competent cells	Novagen	69451

**Chemicals, Peptides, and Recombinant Proteins**		

Cellfectin II	GIBCO	10362100
Pierce protease inhibitor tablet, EDTA-free	Thermo Scientific	A32965
Benzonase	Sigma-Aldrich	E8263
StrepTrap HP	GE Healthcare	28907548
Desthiobiotin	Sigma-Aldrich	D1411
HiTrap Heparin HP	GE Healthcare	17-0407-03
Superose 6 16/70	GE Healthcare	90100042
Superose 6 Increase 10/300	GE Healthcare	29091596
Alexa Fluor 647 C_2_ maleimide	Invitrogen	A20347
Coenzyme A trilithium salt	Sigma-Aldrich	C3019
Sfp transferase	Yin et al. (2006)	N/A
Strep-Tactin Sepharose resin	IBA	2-1201-025
Human condensin I-strep	This paper	N/A
Human condensin II-strep	This paper	N/A
Human condensin I Qloop-strep	This paper	N/A
Human condensin II Qloop-strep	This paper	N/A
Human condensin I-HA-strep	This paper	N/A
Human condensin II-HA-strep	This paper	N/A
Human condensin I-ybbR-strep	This paper	N/A
Human condensin II-ybbR&HA-strep	This paper	N/A
Human condensin I Qloop-ybbR-strep	This paper	N/A
Human condensin II Qloop-ybbR&HA-strep	This paper	N/A
18:1 (Δ9-Cis) PC (DOPC)	Avanti Polar Lipids	850375P-1g
18:1 PEG2000 PE	Avanti Polar Lipids	880130P-25mg
18:1 Biotinyl Cap PE	Avanti Polar Lipids	870273C-25mg
Quartz microscope slide	G. Finkenbeiner	N/A
Lambda DNA	NEB	N3011S
YOYO-1	Invitrogen	Y3601
Streptavidin	Sigma-Aldrich	S4762
ATP	Sigma-Aldrich	A2383
ATPγS	Sigma-Aldrich	A1388
T4 DNA Ligase	NEB	M0202S
PEG8000	Sigma-Aldrich	89510
cOmplete protease inhibitor cocktail	Roche	11697498001
AG 501-X8 mixed bed resin	Bio-Rad	1437424
HiPrep Q Fast Flow 16/10	GE Healthcare	28-9365-43
HiPrep SP Fast Flow 16/10	GE Healthcare	28-9365-44
HiLoad 16/600 Superdex 200 pg	GE Healthcare	28-9893-35
Vivaspin 6, 3,000 MWCO	GE Healthcare	28-9322-96
Amicon Ultra-0.5 mL, 10,000 MWCO	Millipore	UFC501024
ATTO-647N maleimide	ATTO-TEC	AD 647N-41
SnakeSkin dialysis tubing, 10,000 MWCO	Thermo Scientific	68100
TCEP	Sigma-Aldrich	C4706
BSA	Sigma-Aldrich	A8022
100mM ATP solution	GE Healthcare	27-2056-01
ATP [α-^32^P]	PerkinElmer	NEG003X250UC

**Deposited Data**		

Mass spec data	This paper	PRIDE: PXD017531
Condensin I density map	This paper	EMD: 10827
Condensin II density map	This paper	EMD: 10833
Raw data	This paper	https://doi.org/10.17632/24gy83xd8p.1

**Experimental Models: Cell Lines**		

Sf9 cells	GIBCO	11496015
High Five cells	GIBCO	B85502

**Oligonucleotides**		

BioL: 5′Phos-AGG TCG CCG CCC-3Bio	Terakawa et al. (2017)	N/A
DigR: 5′Phos-GGG CGG CGA CCT-3Dig_N	Terakawa et al. (2017)	N/A
BioR: 5′Phos-GGG CGG CGA CCT-3Bio	This paper	N/A
Widom 601 sequence	Lowary and Widom (1998)	N/A
Labeled primers for 183 bp dsDNA	This paper	N/A

**Recombinant DNA**		

pBIG2abc SMC2, 6x His SMC4, CapD2, CapG, CapH 2x strep	This paper	N/A
pBIG2abc SMC2, 6x His SMC4, CapD3, CapG2, CapH2 2x strep	This paper	N/A
pBIG2abc SMC2 Q147L, 6x His SMC4 Q229L, CapD2, CapG, CapH 2x strep	This paper	N/A
pBIG2abc SMC2 Q147L, 6x His SMC4 Q229L, CapD3, CapG2, CapH2 2x strep	This paper	N/A
pBIG2abc SMC2, 6x His SMC4, CapD2, CapG, CapH 3x HA + 2x strep	This paper	N/A
pBIG2abc SMC2, 6x His SMC4, CapD3, CapG2, CapH2 3x HA + 2x strep	This paper	N/A
pBIG2abc SMC2, 6x His SMC4, CapD2, CapG, CapH ybbR + 2x strep	This paper	N/A
pBIG2abc SMC2 Q147L, 6x His SMC4 Q229L, CapD2, CapG, CapH ybbR + 2x strep	This paper	N/A
pBIG2abc SMC2, ybbR SMC4, CapD3, CapG2, CapH2 2x strep	This paper	N/A
pBIG2abc SMC2 Q147L, ybbR SMC4 Q229L, CapD3, CapG2, CapH2 2x strep	This paper	N/A
pLIB CAP-D3	This paper	N/A
pLIB CAP-D3 with C-terminal strep-tag	This paper	N/A
pLIB CAP-G2	This paper	N/A

**Software and Algorithms**		

Fiji	Schindelin et al. (2012)	N/A
MATLAB	MathWorks	N/A
Prism 8	GraphPad	N/A
RELION-3	Zivanov et al. (2018)	N/A
MeroX	Götze et al. (2015)	N/A
xVis	Grimm et al. (2015)	N/A
Chimera	Pettersen et al. (2004)	N/A
Xlink Analyzer	Kosinski et al. (2015)	N/A
PyMol	DeLano (2002)	N/A
PDB2PQR	Dolinsky et al. (2004)	N/A
APBS	Baker et al. (2001)	N/A
Python	Python Software Foundation	N/A
NIS-Elements	Nikon	N/A

**Other**		

Nikon Eclipse inverted microscope	Nikon	TE2000-U
488 nm laser	Coherent Sapphire	N/A
640 nm laser	CrystaLaser	N/A
iXon3 EMCCD	Andor	DU-897E-CSO-#BV
Dual-View splitter	Optical Insights	DV-CC

### Resource Availability

#### Lead Contact

Further information and requests for resources and reagents should be directed to and will be fulfilled by the Lead Contact, Eric C. Greene (ecg2108@cumc.columbia.edu).

#### Materials Availability

All unique/stable reagents generated in this study are available from the Lead Contact without restriction.

#### Data and Code Availability

Crosslinking mass-spectroscopy dataset has been deposited in Proteomics Identification Database (PRIDE: PXD017531). Negative stain electron microscopy dataset has been deposited in Electron Microcopy data bank (EMD: 10827, CI; EMD: 10833, CII). Original uncropped gel images for Figure 1, micrographs for Figure 2, as well as single molecule kymographs and films for Figures 3, 4, 5, 6, and 7 are available at Mendeley (https://doi.org/10.17632/24gy83xd8p.1). All codes are available upon reasonable request.

### Experimental Model and Subject Details

Virus carrying vectors for recombinant human condensins was amplified in Sf9 cells. Recombinant human condensins were expressed in and purified from High Five cells.

### Method Details

#### Expression, purification, and labeling of human condensin complexes

All constructs purified and used are listed (see Key Resources Table). The subunits, sub-complexes, and Q-loop mutations of human condensin I and II were assembled into biGBac vectors (Weissmann et al., 2016). Viral bacmids were generated from biGBac vectors using Tn7 transposition in DH10EMBacY cells, transfected into Sf9 cells using cellfectin II (GIBCO) and resultant virus harvested after 3 days. Virus was further amplified in Sf9 cells and protein expressed in High Five cells, which were harvested by centrifugation 3 days after infection. Cell pellets were resuspended in condensin purification buffer (20 mM HEPES [pH 8], 300 mM KCl, 5 mM MgCl_2_, 1 mM DTT, 10% glycerol) supplemented with 1 Pierce protease inhibitor EDTA-free tablet (Thermo Scientific) per 50mL and 25 U/mL of Benzonase (Sigma) and lysed with a dounce homogenizer followed by brief sonication. Lysate was cleared with centrifugation, loaded on to a StrepTrap HP (GE), washed with condensin purification buffer and eluted with condensin purification buffer supplemented with 5 mM Desthiobiotin (Sigma). Protein containing fractions were pooled, diluted 2-fold with Buffer A (20 mM HEPES [pH 8], 5 mM MgCl_2_, 5% glycerol, 1 mM DTT), loaded on to HiTrap Heparin HP column (GE), washed with Buffer A with 250 mM NaCl, then eluted with buffer A with 500 mM NaCl. Finally, size exclusion chromatography was performed using Condensin purification buffer and a Superose 6 16/70 or increase 10/300 column (GE) (Figure 1C). Purified condensin I and II complexes were analyzed by SDS-PAGE (Figure 1D) and MS/MS, indicating they were the major species in each sample and all subunits were present (Table S1).

Labeled condensin I and II; wild-type and corresponding Q-loop mutants were labeled using the SFP transferase to couple a CoA conjugated Alexa647 (Invitrogen) via a ybbR tag at the C terminus of CAP-H in the case of Condensin I, or in the N terminus of SMC4 in the case of Condensin II. Purification of SFP and labeling was performed as in Yin et al. (2006), except that the conjugation CoA with Alexa647-maleimide was performed in 5-fold excess of Alexa647-maleimide, quenched with DTT in 10-fold excess over Alexa647-maleimide and used directly in reactions. Excess unconjugated material was separated from condensin complexes using size exclusion chromatography with a Superose 6 10/300 column and the presence of all subunits and specific conjugation confirmed by SDS-PAGE (Figures S4A–S4D). EMSAs were performed on labeled material to confirm the fluorophore did not affect DNA binding (Figure S4E).

#### Nucleosomes for Gel Shift Analysis

The Widom 601 (Lowary and Widom, 1998) DNA sequence (147 bp underlined) alone and with 36 bp of linker DNA (183 bp DNA), as follows, was used to make nucleosomes: 5′ATC GAG AAT CCC GGT GCC GAG GCC GCT CAA TTG GTC GTA GAC AGC TCT AGC ACC GCT TAA ACG CAC GTA CGC GCT GTC CCC CGC GTT TTA ACC GCC AAG GGG ATT ACT CCC TAG TCT CCA GGC ACG TGT CAG ATA TAT ACA TCC GAT TAA CGA TGC TGG GCA TAA GCG TGG TTC AAT ACC GGC 3′. DNA was generated using large-scale PCR with in-house *Pfu* polymerase and labeled primers (IDT). The obtained PCR products were pooled, and ethanol precipitated. The pellets were re-dissolved in buffer A (10 mM Tris-HCl [pH 8], 1 mM EDTA [pH 8]) and loaded into a Mono Q 5/50 GL ion-exchange column (GE Healthcare) and eluted with a salt gradient in buffer B (10 mM Tris-HCl [pH 8], 2 M NaCl, 1 mM EDTA [pH 8]). Fractions corresponding to the DNA fragments were assessed by 4%–12% polyacrylamide gel, pooled, followed by ethanol precipitation. The pellet was re-dissolved in buffer A, and stored at −20°C.

Expression, purification and assembly of human histones H2A, H3, H4 and *Xenopus laevis* H2B was performed as described previously (Luger et al., 1999). Briefly, histone proteins were expressed in *E. coli,* and purified from inclusion bodies using cation and anion ion-exchange chromatography, then subsequently lyophilized for long-term storage at −20°C. Individual lyophilized histones were mixed at 1.2 fold excess of H2A and H2B and resuspended in unfolding buffer (20 mM Tris-HCl pH 7.5, 7 M guanidine hydrochloride, 5 mM DTT) for 45 minutes at room temperature, and dialyzed against refolding buffer (10 mM Tris [pH 7.5], 2 M NaCl, 1 mM EDTA, 5 mM β-mercaptoethanol) for 18 h at 4°C, and then subjected to size-exclusion chromatography on a S200 16/60 gel filtration column (GE Healthcare) equilibrated in refolding buffer. The fractions corresponding to histone octamers were pooled, concentrated and flash frozen in liquid nitrogen and stored at −80°C. The nucleosomes were reconstituted by salt dialysis method as described previously (Dyer et al., 2004). Purified DNA and histone octamer were mixed at 1:1.1 ratio of DNA:octamer in a high salt buffer (10 mM Tris-HCl pH 7.5, 2 M NaCl, 1 mM EDTA pH 8, 1 mM DTT) and the sample was dialyzed gradually to low salt buffer (10 mM Tris-HCl pH 7.5, 0.2 M NaCl, 1 mM EDTA pH 8, 1 mM DTT) over 24 h at 4°C using a peristaltic pump. The sample was further dialyzed against in low salt buffer for 4 h, then in nucleosome buffer (20 mM Tris-HCl pH 7.5, 50 mM NaCl, 1 mM EDTA pH 8, 1 mM DTT) overnight at 4°C, before being concentrated and stored at 4°C

#### Gel shift analysis

Double stranded DNA for EMSAs were made by annealing single stranded DNA oligos using a temperature gradient from 95 to 4°C. DNA oligos were purchased with 5′ Cy5 fluorophore or 6-FAM on the reverse strand (IDT), with the 30 bp sequence composed of 5′-CTG TCA CAC CCT GTC ACA CCT GTC ACA C-3′, and the 36 bp sequence was 5′ TAA CGA TGC TGG GCA TAA GCG TGG TTC AAT ACC GGC-3′. EMSAs with unlabeled protein were performed by incubating 50 nM of Cy5 labeled DNA with indicated concentration of protein on ice for ∼30 minutes in condensin purification buffer, before running 4 μL on 2% agarose gel in 0.5x Tris borate buffer (TB) for 30-60 minutes. EMSAs with Alexa647 labeled protein were performed by incubating 25 nM of 6FAM labeled 30 bp DNA with indicated concentration of protein on ice for ∼30 minutes in condensin purification buffer, before running 6 μL on 2% agarose gel in 0.5x TB for 30 minutes. Gels were imaged using a Typhoon FLA 9000 scanner (GE) and analyzed with imageQuant. Nucleosome EMSAs were performed by incubating 50 nM of nucleosome or free dsDNA with indicated concentration of protein and running on 2% Agarose for 60 minutes.

#### ATP hydrolysis Assays

ATP reactions were performed at 37°C using 0.2 μM of protein in ATPase buffer (10 mM Tris [pH 7.5], 100 mM NaCl, 5 mM MgCl_2_, 0.5 mM DTT and 0.1 mg/ml BSA) in the presence or absence of 20 μM of 50 bp of annealed dsDNA. Reactions were started by adding 2 mM of cold ATP supplemented with 1 μCu of [α-^32^P] ATP (800Ci/mmol) (Perkin-Elmer) and aliquots were removed and quenched with 100 mM EDTA at multiple time intervals. Aliquots were spotted onto PEI cellulose F TLC plates (Merck) and run in 1 M formic acid and 300 mM LiCl. TLC plates were used to expose a phosphor-imager plate that was subsequently scanned on a Typhoon FLA 9000 scanner (GE) and analyzed with imageQuant. ATP hydrolysis rate was determined by linear fit of the ADP/ATP ratio during in the linear range of the reaction. ATP hydrolysis assay for each sample was performed with three replicates.

#### Fluorescence polarization

Fluorescence polarization experiments were performed by mixing 50 nM of 6-FAM labeled 30 bp dsDNA with indicated concentrations of protein in FP buffer (20 mM Tris [pH 7.5], 75 mM NaCl, 2.5% glycerol, 1 mM DTT), incubating at room temperature for 30 minutes before reading on a BMG labtech POLARstar Omega plate reader. Three replicates were performed for each protein concentration and globally fit using the following:

$$FP=\left(\frac{FP_{max}}{2.\left[DNA\right]}\right)\left(\left(\left[C\right]+\left[DNA\right]+K_{d}\right)-\sqrt{{\left(\left[C\right]+\left[DNA\right]+K_{d}\right)}^{2}-4.\left[C\right].\left[DNA\right]}\right)$$

Where [C] and [DNA] are the concentration, in μM, of condensin and 6-FAM labeled DNA respectively, *FP*_*max*_ the maximum change in Fluorescence polarization in mAu and *K*_*d*_ is the equilibrium dissociation constant. Fit curves were plotted normalized by dividing by *FP*_*max*_, with error bars indicating standard deviation.

#### Negative Stain Electron microscopy

Samples for EM were prepared using gradient-fixation (Grafix) (Stark, 2010). Gradients were made by sequential freezing of Grafix buffer (50 mM HEPES [pH 8], 750 mM NaCl, 2 mM TCEP, 2 mM MgCl_2_) with 50%, 40%, 30%, 20% and 15% glycerol, and 0.2%, 0.1%, 0.1%, 0.05% and 0% glutaraldehyde respectively. The top three layers (15%–30% glycerol, also contained ∼40 μM of ATPγS). Condensin complexes pre-incubated for ∼30 min with ∼2 mM ATPγS were carefully applied to the top of thawed gradients and run at 29k rpm for up to 20 hours. Fractions containing crosslinked species were quenched with 100 mM Tris pH 8 and SEC run using Superose 6 10/300 column (GE) in EM buffer (20 mM HEPES [pH 8], 200 mM KCl). Negatively stained samples were prepared onto Quantifoil copper grids R1.2/1.3 supporting an additional layer of thin carbon prepared in house. The grids were glow discharged for 1 minute at 15 mA, the sample applied for 10 s and then washed twice with water before staining with 2% uranyl acetate for 1 minute. The grids were screened on a Tecnai T12 electron microscope operating at 120 kV. Micrographs were collected on a Tecnai TF20 (Thermo Fisher Scientific, USA) microscope operating at 200 kV using a 4k x 4k F416 CMOS detector (TVIPS Gmbh, Germany) with the EM-Tools automated data collection software (TVIPS Gmbh, Germany). Example micrographs of condensin I and II are shown (Figure 2A). Data processing was done using RELION-3 (Zivanov et al., 2018).

The model for condensin II was determined first. Particles that were well stained, with obvious density for the coiled coil and heat domains were picked manually, resulting in a total of 3,637 particles. Poor particles were removed with subsequent rounds of 2D classification, resulting in 3,251 particles, which were used for 3D classification. A subset of 1,288 particles were used to generate an initial model. 3D classification was used to generate 3 classes, using the initial model as a reference. One class composed of 1565 particles yielded a model with well defined, domain-like features. After 3D refinement and post processing this model had a resolution of 20.5 Å using FSC 0.143 cut-off criteria and was deposited in the Electron Microcopy data bank (EMD: 10833). The same procedure was followed for condensin I, picking 12,615 particles, with the final 3D class containing 4714 particles and resultant model determined at a resolution of 31.8 Å after post-processing, deposited in the Electron Microcopy data bank (EMD: 10827). The final particle set used for 3D classification was subjected to 2D classification and 2D classes were compared to 3D models, resulting in reasonable agreement in features (Figure 2B). As features of the hinge domain were lost in some 2D classes, a focused classification was performed by moving center of box of aligned particles toward the kink and repeating 2D classification (Figure 2C).

#### Chemical cross-linking and mass spectrometry (XL-MS)

Condensin I and II complexes were diluted in 200 μl buffer containing 20 mM HEPES [pH 8], 300 mM KCl, 10 mM MgCl_2_, 1% glycerol, 1 mM TCEP with addition of 2 mM ATPγS. Disuccinimidyl dibutyric urea (DSBU) was added to the solution to a final concentration of 3 mM and incubated at 25°C for 1 hour. The cross-linking reaction was terminated by adding　Tris-HCl pH 8.0 to a final concentration of 100 mM and further incubation at 25°C for 30 min. Samples were taken before and after cross-linking reaction and examined by SDS-PAGE.

Protein digestion, peptide purification and MS analysis were performed as previously described (Pan et al., 2018). Cross-linked condensin complexes were precipitated by mixing with 800 μl acetone and incubation at −20°C for more than 16 hours. After removal of acetone, protein pellets were dissolved in 8 M urea containing 1 mM dithiothreitol (DTT). Chloroacetamide (5.5 mM) was used to alkylate the cysteine residues. Proteolysis was performed by Lys-C for 3 hours at 25°C and followed by trypsin for 16 hours at 25°C. Trifluoroacetic acid (TFA) was added to the solution to a concentration of 0.2% to stop the digestion. Peptides were purified by size-exclusion chromatography (SEC) using a Superdex Peptide 3.2/300 column (GE healthcare). Four SEC fractions (B8-B5) of each cross-linked sample were collected separately and measured twice by LC-MS/MS as previously described (Pan et al., 2018). Dried peptides were dissolved in water containing 0.1% TFA and were separated on the Ultimate 3000 RSLC nano system. Data were acquired using the Q-Exactive Plus mass spectrometer (Thermo Fisher Scientific) in data-dependent MS/MS mode. For full scan MS, we used mass range of m/z 300−1800, resolution of R = 140000 at m/z 200, one microscan using an automated gain control (AGC) target of 3e6 and a maximum injection time (IT) of 50 ms. Then, we acquired up to 10 HCD MS/MS scans of the most intense at least doubly charged ions (resolution 17500, AGC target 1e5, IT 100 ms, isolation window 4.0 m/z, normalized collision energy 25.0, intensity threshold 2e4, dynamic exclusion 20.0 s).

Program MeroX version 1.6.6.6 (Götze et al., 2015) was used for cross-link identification. In the settings of MeroX, the precursor precision and the fragment ion precision were changed to 10.0 and 20.0 ppm, respectively. RISE mode was used and the maximum missing ions was set to 1. A false discovery rate of 5% was used as the cut-off to exclude the candidates with lower MeroX scores. A mass-deviation range of −4 to 6 ppm was used to further exclude cross-link candidates with mass deviation outside of the range. Non-redundant cross-link lists only contain the identified cross-links with the highest MeroX scores if the same cross-links were identified more than twice and were deposited on the Proteomics Identification Database (PRIDE: PXD017531). The total list of crosslinks for condensin I and II was filtered for those that are present at least 3 times in total and visualized using *xVis* (Figure S2D) (Grimm et al., 2015).

#### Structural Modeling

To generate a pseudo atomic model, the crystal structure of the human SMC2/4 hinge (PDB: 4U4P, SMC2 residues 507-673, SMC4 610-754) was combined with homology models of SMC2/4 ATPase domain (SMC2 residues 2-199 and 1002-1175, template: *Ct* SMC2 6QJ1 and SMC4 residues 79-286 and 1149-1282, template: *Ct* SMC4 6QJ2), CAP-D3 (residues 18-1063, template: *Ct* Ycs4 6QJ4), CAP-G2 (residues 275-1111, template: human CAP-G 5OQQ), and CAP-H2, (residues 492-574, template *Ct* Brn1 6QJ4) generated using Phyre2 (Kelley et al., 2015) and coiled coils built using CCBuilder2.0, using a radius of 5 nm, a pitch of 70 and an interface angle of 45 degrees (SMC2 residues 200-504 and 673-1001, and SMC4 residues 301-589 and 766-1087). ATPase heads were modeled together in the engaged conformation by aligning above mentioned homology models to ATPase domains in Rad50 crystal structure 5F3W (Liu et al., 2016).

Subunits were sequentially fit into condensin II map using *Chimera* (Pettersen et al., 2004), subtracting the fit density before the subsequent subunits were fit. The SMC2/4 ATPase heads with CAP-H2 C terminus were fit first, resulting in two likely fits in the density proximal to the coiled-coil region, as the SMC2/4 model had pseudo symmetry. Both possible conformations were built. Next, the coiled-coil domain was fit, such that there would be sufficient space for un-modeled regions between the ATPase domains and the coiled-coils. This arrangement suggests there would be have to be considerable bend in the coiled-coils near the ATPase heads. This region of SMC arms can contain a number of glycine and proline residues, which cause a break in helicity, demonstrated in the crystal structure of the *S. cerevisiae* SMC3 ATPase (PDB: 4UX3) and *B. subtilis* SMC 5NMO and has been referred to as the “joint” (Diebold-Durand et al., 2017). Of the residual unaccounted map, one region had harp-shaped density, which was complementary for the shape of the CAP-G2 homology model. The remaining density was fit with the CAP-D3 model, which places the N terminus of CAP-D3 near the coiled-coil region of SMC2 and the C terminus near CAP-G2.

While there was not sufficient structural information to model the remaining CAP-H2, the trajectory can be traced based on homology with *Ct* Brn1 (Figures S1E–S1H) the modeled subunit arrangement is consistent with interaction studies indicating the N terminus of CAP-H2 binds to SMC2, then to CAP-D3, then CAP-G2 and has the C terminus interacting with SMC4 (Onn et al., 2007).

#### Structural Analysis

The condensin II model was then compared to mass-spec crosslinking data. Using *Xlink Analyzer* (Kosinski et al., 2015) with a distance cut-off threshold of 35 Å. The model presented in text satisfied 83% of crosslinked pairs present in the modelled region, was consistent with unique crosslinks between the N terminus of CAP-D3 with the N terminus of CAP-H2, and with what is known from homology, as the kleisin CAP-H2 N terminus interacts with SMC2 before running parallel down CAP-D3, hence was considered to be more likely than the alternative arrangement if SMC2 and 4 were swapped. The crosslinking data also supports the orientation of CAP-G2 and a considerable interaction between the N-terminal region of CAP-G2 and the C-terminal region of CAP-D3, as the crosslink from CAP-G2:340 to CAP-D3:1214 is the most abundant non-SMC inter-domain crosslinks. The engagement of the ATPase heads is also supported by crosslinks, such as those between SMC2:1079-SMC4:1267 and SMC2:12-SMC4:1187. There are multiple crosslinks between the SMC2/4 coiled-coils, and these are mostly enriched in region between the kink to the hinge, suggesting this region may close independently of the rest of the SMC arms. Examining the violated crosslinks, there is a cluster of violated crosslinks between the interface of CAP-G2 with SMC2, and a crosslink between the hinge and coiled-coil region near the ATPase domain, which could represent an alternate conformation of the complex. All intra-subunit cross links in the “proboscis” (helical extension of HEAT repeat motif 10) were violated, suggesting this feature of *Ct* Ycs4 is either not present or not present in the same position of human CAP-D3 (Hassler et al., 2019).

Electrostatic surface potential was determined using *PDB2PQR* (Dolinsky et al., 2004) and *APBS* (Baker et al., 2001), and visualization performed in *Pymol* (DeLano, 2002).

#### Pull Down

Virus for CAP-D3 with or without a Strep-tagII and CAP-G2 were used to co-infect High Five insect cells. Infected cells were harvested after 3 days, cell pellets were resuspended in 20 mM HEPES [pH 8], 200 mM NaCl, 1 mM DTT, 10% glycerol), lysed with sonication and incubated with 100 U/mL of Benzonase and 1mM MgCl_2_. Lysate was cleared with centrifugation and incubated with Strep-Tactin Sepharose resin (IBA). Resin was washed 5 times with lysis buffer and resin eluted with 1x NuPAGE LDS loading buffer. Sample analyzed with SDS-PAGE and stained with Coomassie. Samples were diluted for analysis by western blotting using antibodies against CAP-D3 (Santa Cruz, sc-81597) and CAP-G2 (Bethyl, A300-605A) and developed with DyLight 800 florescent secondary antibodies (Cell Signaling Technology, 5151 and 5257).

#### Single-molecule DNA curtain

Single-molecule DNA curtain assays were performed as previously described (Greene et al., 2010, Terakawa et al., 2017). To prepare DNA substrates for curtain experiments, 1.6 pmol of λ-DNA (N3011S, New England Biolabs) was mixed with 100-fold molar excess of biotin- or digoxigenin end-modified oligonucleotides (IDT) in 1X T4 ligation buffer (B0202S, New England Biolabs) in a 600 μL reaction. Oligos BioL (5′Phos-AGG TCG CCG CCC-3Bio) and DigR (5′Phos-GGG CGG CGA CCT-3Dig_N) were used for substrate in single- and double-tethered experiments, whereas BioL and BioR (5′Phos-GGGCGGCGACCT-3Bio) were used to generate the U-shaped DNA substrate. The mixture was assembled at 65°C and incubated for 5 minutes before cooled down to room temperature. 5 μL of T4 DNA ligase (M0202S, New England Biolabs) was then added to the reaction and ligation was carried out overnight at room temperature. DNA substrate was precipitated by adding PEG8000 (Sigma, Cat. No. 89510) and MgCl_2_ to the final concentrations of 10% and 10 mM, respectively, and incubating at 4°C with rotation. Following centrifugation of sample at 14,000 x *g* for 5 minutes, resulting DNA pellet was washed once with 70% ethanol, resuspended in ddH_2_O, and stored at 4°C. Briefly, in a custom-made microfluidic flow cell, biotinylated DNA substrate was anchored to the supported lipid bilayer on the surface of a quartz slide with nanofabricated chromium barriers, through biotin-streptavidin linkage. Double-stranded DNA was stained with buffer containing 0.5 nM YOYO-1 (Y3601, Invitrogen) and visualized through 488 nm excitation (Coherent Sapphire) on a custom-built prism-type TIRF microscope (Nikon Eclipse TE2000-U) equipped with a 60x water immersion objective and an EMCCD camera (Photometrics Cascade II: 512 or Andor iXon X3). All experiments were carried out at 37°C in condensin buffer (40 mM Tris-HCl [pH 7.5], 125 mM NaCl, 5 mM MgCl_2_, 1 mM DTT, 0.5 mg/mL BSA), supplemented with either 4 mM ATP or ATPγS. Unlabeled condensin in condensin buffer was continuously injected into the flow cell via a syringe pump (KD Scientific) at the rate of 0.1 mL/min as image frames were acquired. In single-tethered experiments, the biotinylated ends of DNA were aligned at the barriers while the free ends were extended by flow. In U-shaped DNA experiments, both ends of the DNA molecules were anchored to the lipid, and the molecule adopted a ‘U’ shape under continuous flow when its anchor points were aligned in adjacent wells of the chromium barrier. To double-tether DNA, flow cell containing DNA was washed with condensin buffer at the rate of 0.125 mL/min for 20 minutes. During this time period, the Dig-modified end of DNA was extended above chromium pedestals coated with anti-Digoxigenin antibody (Roche, 11214667001), which were located 8 μm from the barriers. Buffer flow was subsequently turned off during data collection, where DNA remained extended at 50% of its 16 μm contour length. Additionally, in experiments where nucleosome-bound DNA was used, DNA was first injected into the flow cell in salt-free nucleosome buffer (40 mM Tris-HCl [pH 7.5], 1 mM DTT, 0.5 mg/mL BSA), before the nucleosome buffer was rapidly flushed out and replaced with condensin buffer at the rate of 1 mL/min. Dual color imaging of YOYO1-DNA and either ATTO-647N-labeled nucleosomes, or Alexa647-labeled condensin was carried out through additional 640 nm laser excitation (CrystaLaser) and a Dual-View splitter (Optical Insights), allowing simultaneous acquisition in both channels. Photobleaching of CI-Alexa647 was achieved via continuous illumination of the 640 nm laser.

#### Single-molecule data analysis

ND2 files or TIFF image series were imported in Fiji (Schindelin et al., 2012) and saved as TIFF stacks before processing. For single-tethered DNA compaction experiments, a kymograph is generated for each DNA molecule for analysis. Average compaction velocities were estimated by calculating slopes of line segments approximating the trajectory of the DNA free end in the kymograph. Compaction events by definition had positive slopes (velocities). Slippage or disruption of compaction, as well as other events where the DNA free end moved away from the barrier, therefore had negative velocities and were discarded in plotting. Processivity is defined as the total positive displacement between events with negative slopes, or the beginning or end of the trajectory. Lag time in compaction initiation is defined as the elapsed time between the arrival of condensins in the sample chamber and the beginning of DNA compaction events. A compaction event whose observed processivity was considered censored if said event was terminated due to protein dissociation, DNA breakage, free end coming within 1 pixel of the barrier position, or end of data collection period. Survival function of processivity was estimated with the Kaplan-Meier estimator, using the Seaborn library in Python.

Symmetry of U-shaped DNA looping events was determined in two ways. In the first pass, each looping event was carefully examined visually. A trajectory was classified as symmetric if the increasing DNA signal punctum remained on the central symmetry axis of the U-shape as it progressed toward the barrier, or asymmetric If the progression visibly veered toward one side or the other. All analyzed events were unambiguously classified by these criteria. In a second pass, in order to quantitatively confirm the classification, the lengths of DNA segment on either side of the moving punctum were measured frame-by-frame for each looping event. The time series of the ratios of these two lengths was then fitted to a linear line, over a normalized reaction coordinate from 0 to 1. A symmetric event, where the lengths of DNA on either side of the punctum decrease at roughly the same rate would yield a fitted line with slope ∼0. An asymmetric event, where the length of DNA on one side decreases significantly faster than that on the other side would yield a fitted line of slope >>0 or <<0. Analysis of fitted parameters randomly drawn from half of the condensin I events previously classified as symmetric revealed that the mean and standard deviation of the fitted slope are ∼0.03 and 0.07, respectively. Applying the criterion that slopes outside ± 5 standard deviations of the mean would be classified asymmetric, this second pass on the rest of the dataset including both labeled and unlabeled condensin I and II resulted in the same classification as before, validating the robustness of the visual approach. In control experiments with yeast condensin, all events (n = 77/77) had been visually classified as asymmetric. Application of the same quantitative analysis further confirmed previous classifications.

In U-shaped DNA looping experiments, average velocities of symmetric looping events were measured by estimating the rate at which the looped, overlapping portion of DNA increased in length. Average velocities of asymmetric looping events were measured by manually tracking the displacement of the bright looped DNA signal over time. The same procedure was also employed to obtain looping velocities in control experiments using yeast condensin. Values obtained were also consistent with previously published results using DNA double-tethered to the surface (Ganji et al., 2018).

All distances were initially measured in number of pixels and subsequently converted to μm with the conversion factor of 0.267 μm/pixel, based on the combination of the sensor pixel size and 60X objective. Under the single-molecule experimental conditions specified above, the average extensions of YOYO1-stained naked DNA, nucleosome-bound DNA, and U-shaped DNA were 10.75 ± 0.30 μm (n = 294), 9.11 ± 0.44 μm (n = 112), and 9.54 ± 0.55 μm (n = 91), respectively. Apparent velocities and processivities were converted from physical quantities (μm/s and μm) to more biologically relevant units (bp/s and bp) by assuming that the entire contour length of the DNA (48,502 bp) was uniformly mapped onto the measured average end-to-end extension. This conversion was carried out only to facilitate comparisons with previously reported values. It is important to note that the effective conversion factors (bp/μm) depend not only on the flow rate, but also the DNA geometry, among other factors. Velocity values are reported as median (interquartile range, or IQR) throughout.

#### Histone purification and nucleosome reconstitution

*Xenopus* H2A (wild-type), H2B (V119C), H3 (C110S), or H4 (wild-type) were expressed in *E. coli* BL21 (DE3) pLysS cells (Novagen, Cat. No. 69451), individually, using a pET3A-based plasmid. Cultures (6 L for each histone) were grown to OD600 ∼0.6 at 37°C, and protein expression was induced with 0.5 mM IPTG for 4 hours at 37°C. Cells were harvested at 23°C by centrifugation (3000xg for 10 minutes) and resuspended into 40 mL buffer containing 50 mM Tris (pH 8.0), 10% (w/v) sucrose, 1 mM TCEP, 1 mM PMSF, 1X cOmplete™ Protease Inhibitor Cocktail (Roche, 11697498001) and frozen at −80°C. Thaw the cells on a warm water bath and added 40 mL Tris-washing buffer containing 50 mM Tris [pH 7.5], 100 mM NaCl, 5 mM β-mercaptoethanol, 1mM EDTA, 1X cOmplete™ Protease Inhibitor Cocktail, 1% w/v Triton X-100. Cell suspensions were sonicated for 2 minutes (10 s on and 50 s off) at 60% power output. Inclusion bodies were harvested by centrifugation at 20,000xg for 20 minutes at 4°C. The protein pellets were then washed by completely resuspending and spinning 4 times with Tris-washing buffer; Triton X-100 was omitted from the Tris-washing buffer for the final two washes. After the last wash, pellets were stored at −80°C until use. Pellets were mixed with 1 mL dimethyl sulfoxide (DMSO) for 30-60 min at room temperature before adding 40 mL unfolding buffer (20 mM Tris (pH 7.5), 7 M guanidinium-HCL, 10 mM DTT) with stirring for 1 hour at room temperature. Then, suspensions were centrifuged at 20,000xg for 20 minutes to remove any remaining insoluble material. The supernatant was dialyzed against 2L of urea buffer (10 mM Tris (pH 8.0), 7 M urea, 1 mM EDTA, 5 mM β-mercaptoethanol and either 100 mM NaCl for H2A and H2B, or 200 mM NaCl for H3 and H4) in dialysis tubing (3,500 MWCO) overnight at 4°C with two buffer changes. Prior to use, urea buffer was deionized using AG 501-X8 resin (Bio-Rad, Cat. No. 1437424) for 2 hours at room temperature. The dialyzed histones were purified with an Akta FPLC system (GE Healthcare) by loading onto a tandem HiPrep Q Fast Flow 16/10 followed by a HiPrep SP Fast Flow 16/10 column (GE Healthcare) pre-equilibrated with urea buffer at a flow rate of 0.2 mL/min. The columns were washed with more than 75 mL of urea buffer (100 mM NaCl for H2A and H2B, 200 mM NaCl for H3 and H4), the HiPrep Q FF column was then removed, and the histones bound to the HiPrep SP FF were eluted in urea buffer at 0.2 mL/min using a NaCl gradient to 0.5 M over a total volume of 100 mL. Fractions contain histone were combined and dialyzed against 4 L buffer containing 10mM Tris (pH8.0) and 5 mM β-mercaptoethanol using 3,500 MWCO dialysis tubing with four buffer changes. The initial two rounds were conducted overnight at 4°C, followed by the finial two buffer changes without β-mercaptoethanol with 6-hour intervals. Finally, the histones were frozen in liquid nitrogen and lyophilized in a Labconco FreeZone 1 lyophilizer for 48 hours. The lyophilized histones were stored at −20°C prior to use.

Lyophilized histones (∼5 mg for each histone) were dissolved into 1 mL unfolding buffer with gentle agitation for 2 hours at room temperature. Histones were mixed at equimolar ratios (use 10%–15% more of H2A/H2B relative to H3/H4) and the mixture was diluted to 1 mg/ml with unfolding buffer, and dialyzed against TEB2000 (10 mM Tris [pH 8.0], 1 mM EDTA, 5 mM β-mercaptoethanol, 2 M NaCl) for 48 hours with 4 buffer changes. The dialyzed histones were then concentrated to 1 mL using a 3,000 MWCO spin concentration device (Vivaspin 6, GE Healthcare), and purified on a HiLoad 16/600 Superdex 200 pg size exclusion column (GE Healthcare) pre-equilibrated with TEB2000 at a flow rate of 1 mL/min. Fractions contain octamer were combined and concentrated. Equal volume of 100% glycerol was added to the purified octamer solutions (final concentration of 50% glycerol) and stored at −20°C.

Prior to the labeling reaction, potential disulfide bonds in reconstituted histone octamers (H2B V119C) were broken down by incubation with 1 mM TCEP for 20 minutes at room temperature. TECP was then removed from the sample using an Amicon Ultra-0.5 centrifugal filter unit (10,000 MWCO, Millipore Sigma UFC501024) with three ∼400-450 μL washes of TE2000 buffer (10 mM Tris [pH 7.0], 1 mM EDTA, 2 M NaCl). 5X molar excess of ATTO-647N maleimide (ATTO-TEC, Cat. No. AD 647N-41) was added to the octamers, and the labeling reaction was incubated at room temperature for 2 hours in the dark. Excess free dye was removed by washing with TE2000 buffer at least 5 times or until flow-through was clear of dye using an Amicon column. Labeling efficiency was determined by both measurement of absorbance (A280) on a NanoDrop 2000 (Thermo Scientific), as well as quantification of SDS-PAGE band intensities scanned on a Typhoon FLA900 (GE Life Sciences) with appropriate settings for fluorescence excitation and emission.

In a 30 μL reconstitution reaction, modified λ-DNA substrate (1.6 nM final concentration) for DNA curtain experiments (BioLDigR for single-tethered and BioLBioR for U-shaped) was mixed with ATTO-647N-labeled histone octamers in TEB2000 buffer with an appropriate DNA:octamer ratio which was adjusted empirically to produce the desired number of nucleosomes per DNA molecule. The reaction was transferred to a home-made dialysis device with 10,000 MWCO SnakeSkin dialysis tubing (Thermo Scientific, Cat. No. 68100) and placed in a beaker containing 100 mL TEB2000 with gentle agitation. Dialysis was carried out overnight at 4°C with TEB buffer (10 mM Tris [pH 8.0], 1 mM EDTA, pH 8.0, 5 mM β-mercaptoethanol) being continuously added to the beaker at the rate of 0.5 mL/min via a peristaltic pump. After the concentration of NaCl drops below 400 mM, the sample was then dialyzed against 100 mL TEB buffer for another 2 hours. Finally, the reconstitution reaction was retrieved from device and stored at 4°C in the dark.

### Quantification and Statistical Analysis

Statistical details of experiments can be found in figure legends or the STAR Methods section.

## Supplemental Information

Document S1. Table S1 and Figures S1–S6

Document S2. Article plus Supplemental Information
