# Supplementary material for: Human Condensin I and II Drive Extensive ATP-Dependent Compaction of Nucleosome-Bound DNA
Source: Mol Cell. 2020 Jul 2;79(1):99–114.e9. doi: 10.1016/j.molcel.2020.04.026 (PMC7335352; doi:10.1016/j.molcel.2020.04.026)
Supplement: Document S1. Table S1 and Figures S1–S6 [file mmc1.pdf]

Molecular Cell, Volume *79*

Supplemental Information

Human Condensin I and II Drive Extensive ATP-Dependent Compaction of Nucleosome-Bound DNA

Muwen Kong, Erin E. Cutts, Dongqing Pan, Fabienne Beuron, Thangavelu Kaliyappan, Chaoyou Xue, Edward P. Morris, Andrea Musacchio, Alessandro Vannini, and Eric C. Greene

**Condensin I sample**			
**Identified Proteins**	**Accession Number**	**Molecular Weight**	**Unique peptide count**
Condensin complex subunit 1	CND1_HUMAN	157 kDa	98
Structural maintenance of chromosomes protein 4	SMC4_HUMAN	147 kDa	108
Condensin complex subunit 3	CND3_HUMAN	114 kDa	75
Structural maintenance of chromosomes protein 2	SMC2_HUMAN	136 kDa	98
Condensin complex subunit 2	CND2_HUMAN	83 kDa	43
Trypsin	TRYP_PIG	24 kDa	5
Condensin complex subunit 1	CND1_XENLA	154 kDa	2
RuBisCO large subunit-binding protein subunit alpha, chloroplastic	RUB2_BRANA	62 kDa	2
Condensin complex subunit 1	CND1_MOUSE	156 kDa	1
Structural maintenance of chromosomes protein 2	SMC2_XENLA	136 kDa	2
UPF0747 protein BPUM_1405	Y1405_BACP2	63 kDa	1
**Condensin II sample**			
**Identified Proteins**	**Accession Number**	**Molecular Weight**	**Unique peptide count**
Condensin-2 complex subunit G2	CNDG2_HUMAN	131 kDa	82
Structural maintenance of chromosomes protein 4	SMC4_HUMAN	147 kDa	97
Condensin-2 complex subunit D3	CNDD3_HUMAN	169 kDa	90
Structural maintenance of chromosomes protein 2	SMC2_HUMAN	136 kDa	75
Condensin-2 complex subunit H2	CNDH2_HUMAN	68 kDa	32
Tubulin alpha-3C/D chain	TBA3C_HUMAN	50 kDa	5
Trypsin	TRYP_PIG	24 kDa	5
Tubulin beta-1 chain	TBB1_MANSE	50 kDa	4
Heat shock 70 kDa protein cognate 4	HSP7D_MANSE	71 kDa	3
Chaperone protein DnaK	DNAK_LYSSC (+1)	65 kDa	2
Heat shock protein 70 A1	HSP71_ANOAL (+1)	70 kDa	2
RuBisCO large subunit-binding protein subunit alpha, chloroplastic (Fragment)	RUB1_BRANA (+1)	58 kDa	2

Table S1. Mass-Spectrometry Analysis of Purified Recombinant Human Condensins I and II, Related to Figures 1 and 2

Figure S1

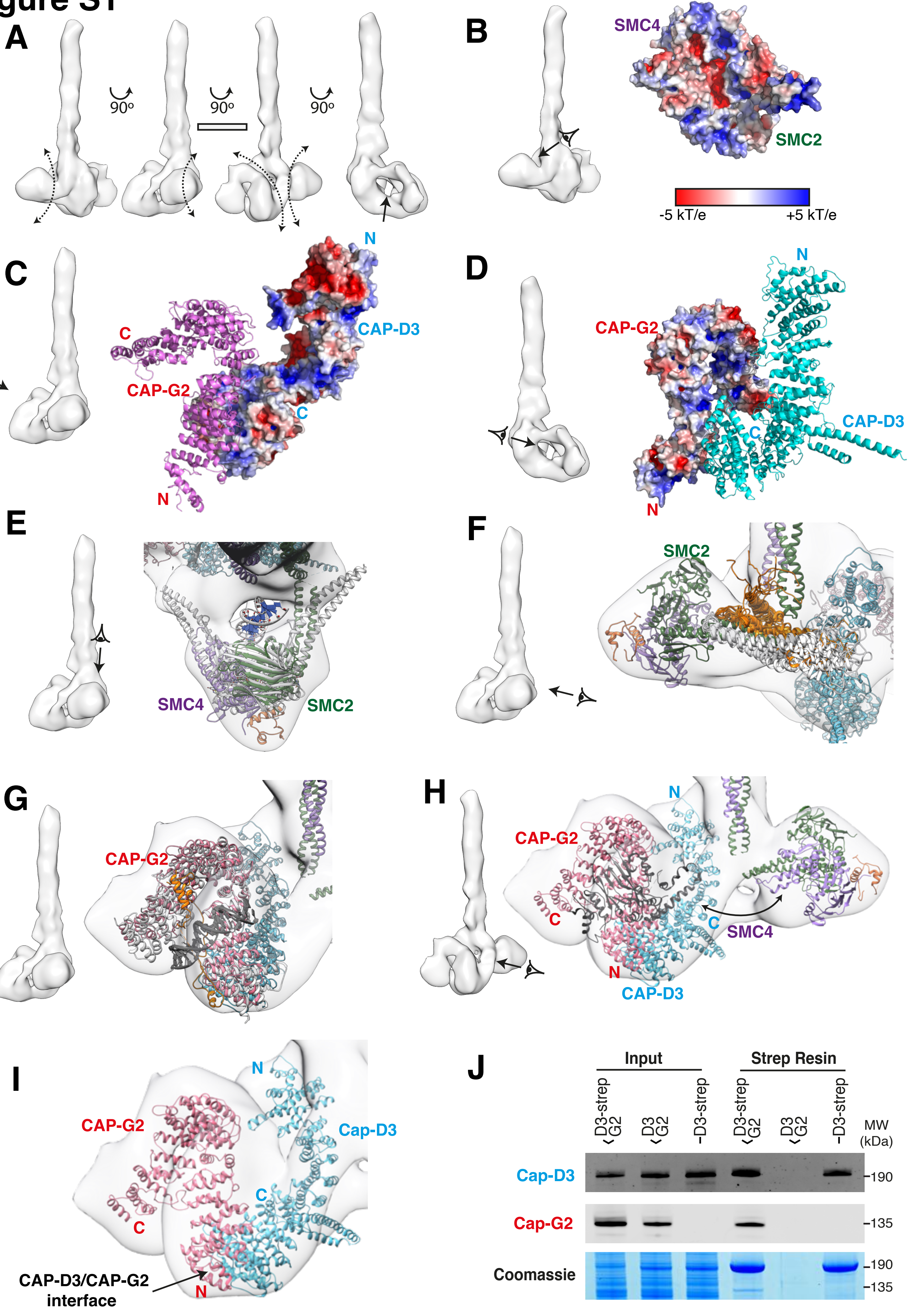
A
90°
90°
90°
B
SMC4
SMC2
-5 kT/e
+5 kT/e
C
N
C
CAP-D3
CAP-G2
C
N
D
N
CAP-G2
CAP-D3
C
N
E
SMC4
SMC2
F
SMC2
G
CAP-G2
H
N
CAP-G2
C
C
SMC4
N
CAP-D3
I
N
CAP-G2
Cap-D3
C
C
N
CAP-D3/CAP-G2
interface
J
Input
Strep Resin
D3-strep
G2
D3
G2
D3-strep
D3-strep
G2
D3
G2
D3-strep
MW
(kDa)
Cap-D3
190
Cap-G2
135
Coomassie
190
135

Figure S1. Possible DNA Entrapment Compartments Suggested by Condensin II Model, Related to Figure 2

(A) 3D model of condensin II, with arrows indicating possible DNA entrapment compartments (scale bar is 10 nm).

(B) Electrostatic potential map of interface between SMC2 and SMC4 ATPase domain.

(C) Electrostatic potential map of CAP–D3 model, with adjacent CAP–G2 model.

(D) Electrostatic potential map of CAP–G2 model, with adjacent CAP–D3 model.

(E) Overlay of condensin II model with Ct Rad50 DNA bound structure (5DAC), which places bound DNA where the hole in density map is located.

(F) Overlay of condensin II model and NMR structure of N-terminal Brn1/SMC2 coiled-coil fusion (PDB: 6Q6E).

(G) Overlay with S. cerevisiae Ycg1/Brn1 structure (PDB: 5OQN) with modelled CAP-G2.

(H) Overlay of Ct Ycs4/Brn1/SMC4 ATPase with modelled CAP-D3. Insert in all figures indicates direct of view of the overlay. In all figures, SMC2 is green, SMC4 is purple, CAP-D3 is light blue, CAP-G2 is pink and CAP-H2/Brn1 is orange. Homologous proteins, except Brn1, are shown in grey.

(I) Focus of modelled CAP-D3 and CAP-G2, indicating the potential interaction between these domains.

(J) Pull-down assays using insect cell lysates co-expressing CAP-D3 and CAP-G2, indicating that CAP-D3 strep can pull-down CAP-G2, while the control using CAP-D3 without a strep tag does not pull-down CAP-G2.

Figure S2

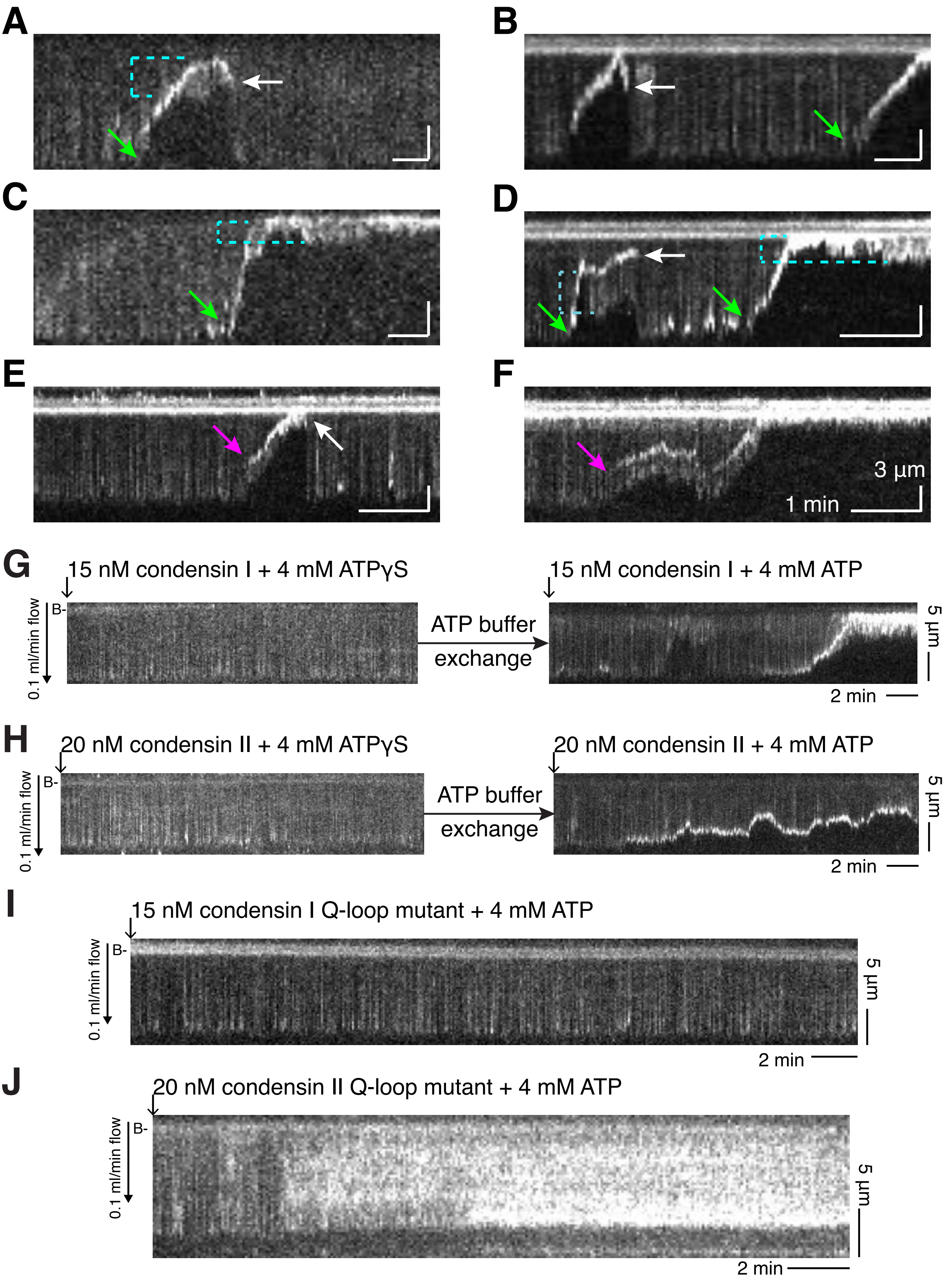
A
B
C
D
E
F
3 µm
1 min
G
15 nM condensin I + 4 mM ATPγS
B-
0.1 ml/min flow
ATP buffer
exchange
15 nM condensin I + 4 mM ATP
5 µm
2 min
H
20 nM condensin II + 4 mM ATPγS
B-
0.1 ml/min flow
ATP buffer
exchange
20 nM condensin II + 4 mM ATP
5 µm
2 min
I
15 nM condensin I Q-loop mutant + 4 mM ATP
B-
0.1 ml/min flow
5 µm
2 min
J
20 nM condensin II Q-loop mutant + 4 mM ATP
B-
0.1 ml/min flow
5 µm
2 min

Figure S2. Representative Kymographs of Single-Tethered DNA Compaction by Human Condensins, Related to Figure 3

(A) to (F) Representative kymographs of human condensin behavior during compaction of single-tethered DNA. Green and magenta arrows indicate compaction events that initiated from either the free end or within the internal portion of the DNA molecule, respectively. White arrows indicate sudden and complete release of compacted DNA to its full length. Cyan brackets indicate compacted DNA loops extended by flow.

(G) and (H) Representative kymographs of DNA showing no compaction by condensin I or condensin II in the presence of 4 mM ATPγS. After buffer exchange, these same DNA molecules were readily compacted by condensin I and condensin II in the presence of 4 mM ATP.

(I) and (J) Representative kymographs of DNA showing no compaction by Q-loop mutants of condensin I or condensin II, in the presence of 4 mM ATP. Note that CII Q-loop mutant was extremely prone to nonspecific interactions with the surface, hence the aberrant appearance of DNA; the brighter signal reflects adherence to the sample chamber surface.

Figure S3

A

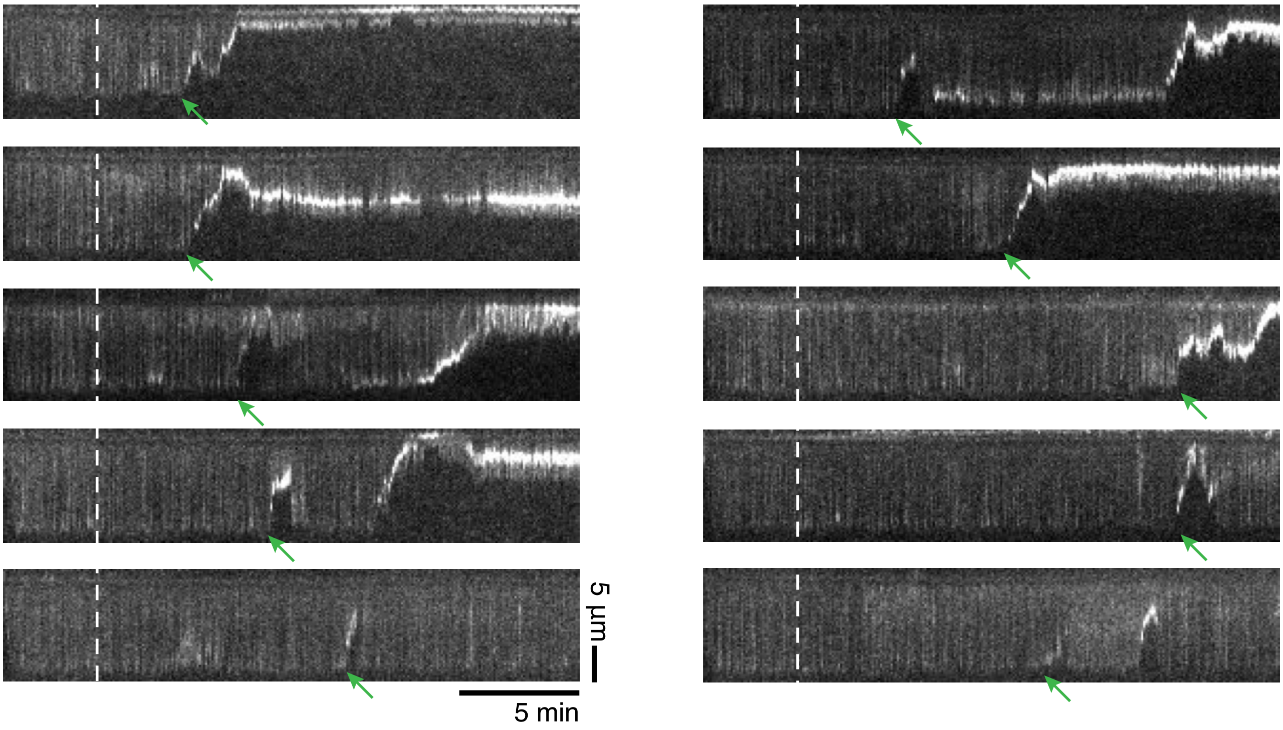

B

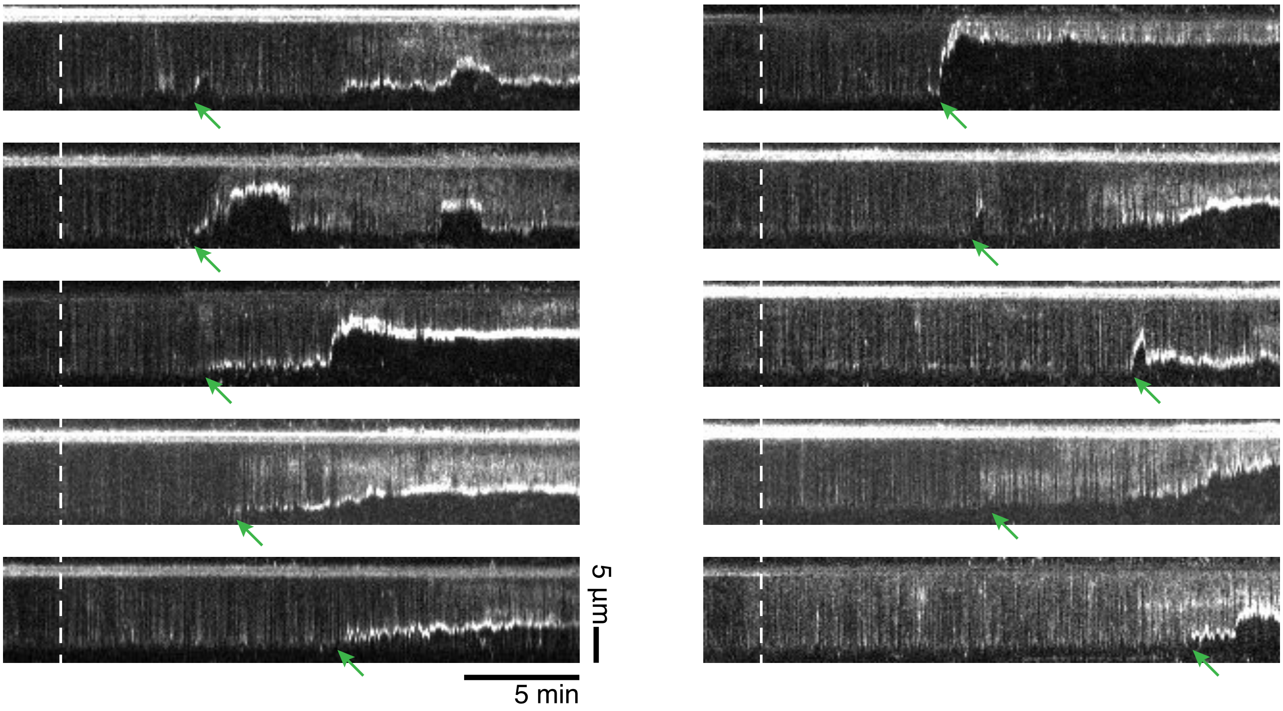

Figure S3. Stochastic Initiation of Compaction, Related to Figure 3

(A) and (B) Representative kymographs of YOYO1–stained single–tethered DNA showing a wide range of initiation start times in compaction events by condensin I and II, respectively. White dashed lines mark the proteins' predicted times of arrival in sample chamber. Green arrows indicate initiations of the first compaction events on each DNA molecule.

Figure S4

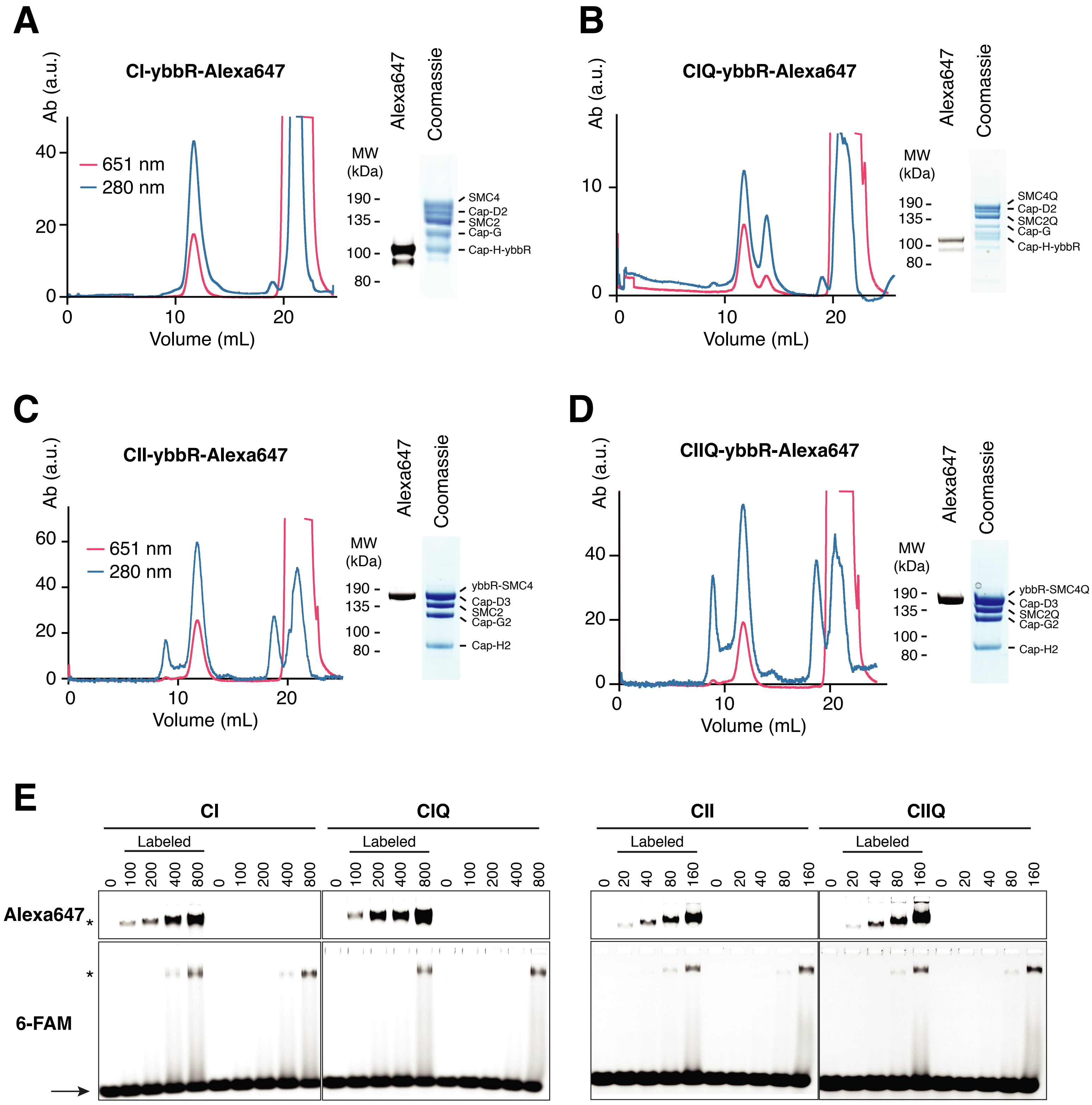

A
CI-ybbR-Alexa647
Ab (a.u.)
651 nm
280 nm
Volume (mL)
Alexa647
Coomassie
MW (kDa)
SMC4
Cap-D2
SMC2
Cap-G
Cap-H-ybbR
B
CIQ-ybbR-Alexa647
SMC4Q
SMC2Q
C
CII-ybbR-Alexa647
ybbR-SMC4
Cap-D3
Cap-G2
Cap-H2
D
CIIQ-ybbR-Alexa647
ybbR-SMC4Q
E
CI
CIQ
CII
CIIQ
Labeled
Alexa647
6-FAM

Figure S4. Purification of Alexa647-labeled Condensin I and II, Related to Figure 4

(A) Chromatogram of gel filtration with Superose 6 Increase 10/300 column of labeled CI, showing absorption at the excitation wavelength of Alexa647 (651 nm) and protein absorption (280 nm). Protein peak elutes at ~11 mL, while excess SFP and unconjugated fluorophore elutes after 20 mL. Insert shows SDS page sample from protein peak, imaged for Alexa647, followed by Coomassie staining.

(B) to (D) As in (A) for CI Q loop mutant, CII and CII Q loop mutant respectively.

(E) EMSA assay using 6-FAM labeled 30bp DNA, and Alexa647 labeled and unlabeled CI, CIQ, CII and CIIQ from left to right respectively. Arrow indicates free DNA, * indicates shifted DNA.

Figure S5

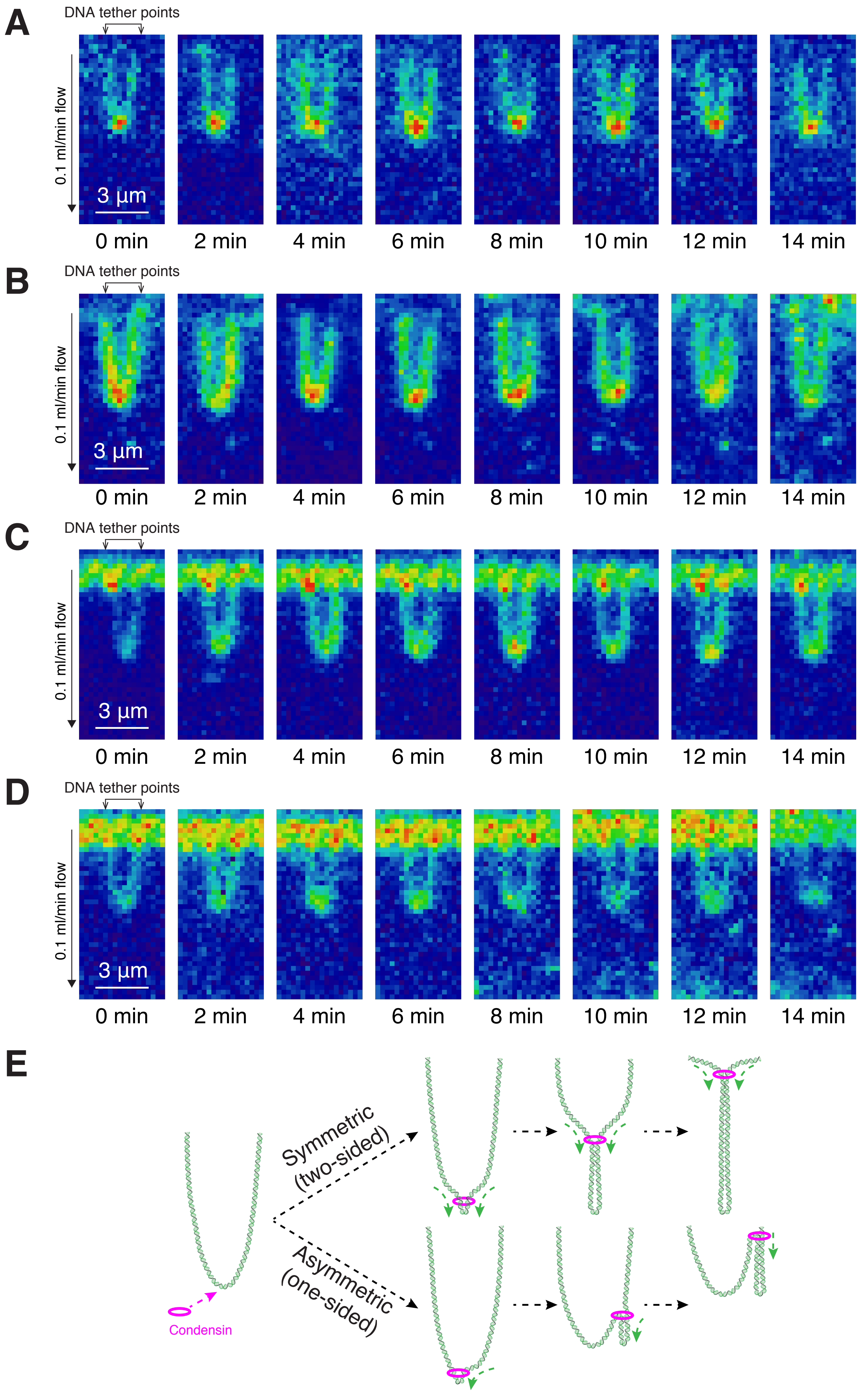

A
DNA tether points
0.1 ml/min flow
3 µm
0 min
2 min
4 min
6 min
8 min
10 min
12 min
14 min
B
DNA tether points
0.1 ml/min flow
3 µm
0 min
2 min
4 min
6 min
8 min
10 min
12 min
14 min
C
DNA tether points
0.1 ml/min flow
3 µm
0 min
2 min
4 min
6 min
8 min
10 min
12 min
14 min
D
DNA tether points
0.1 ml/min flow
3 µm
0 min
2 min
4 min
6 min
8 min
10 min
12 min
14 min
E
Symmetric (two-sided)
Asymmetric (one-sided)
Condensin

Figure S5. Looping and Compaction of U-shaped DNA Requires ATP Hydrolysis, Related to Figure 5

(A) and (B) Representative snapshots of U-shaped DNA showing no looping or compaction by condensin I (n = 0/79) or condensin II (n = 0/73) in the presence of 4 mM ATPγS.

(C) and (D) Representative snapshots of U-shaped DNA showing no looping or compaction by Q-loop mutants of condensin I (n = 0/67) or condensin II (n = 0/59), in the presence of 4 mM ATP.

(E) Schematics of symmetric (two-sided) and asymmetric (one-sided) loop extrusion of U-shaped DNA.

Figure S6

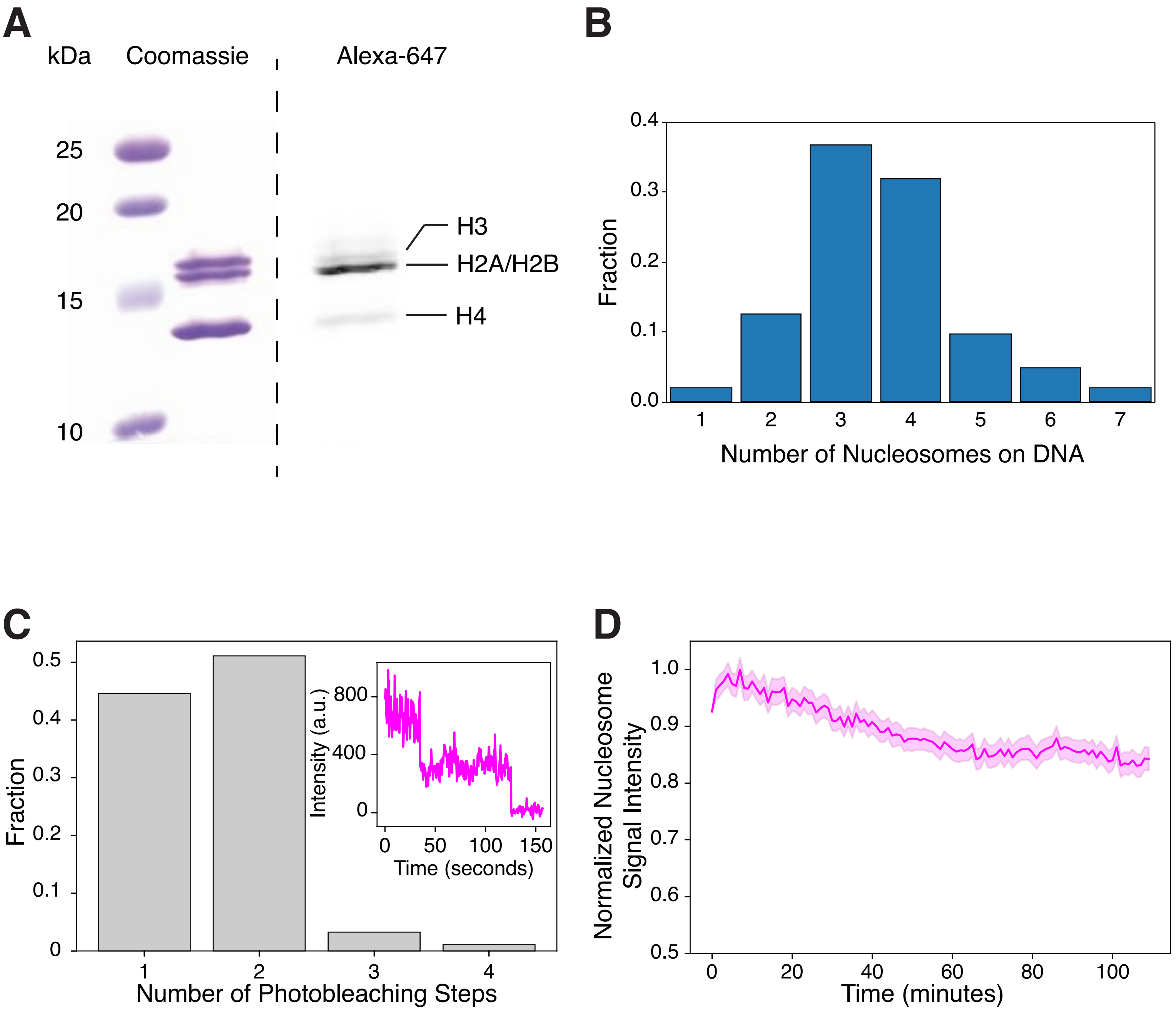
A
kDa
Coomassie
Alexa-647
25
20
15
10
H3
H2A/H2B
H4
B
Fraction
0.0
0.1
0.2
0.3
0.4
1
2
3
4
5
6
7
Number of Nucleosomes on DNA
C
Fraction
0
0.1
0.2
0.3
0.4
0.5
1
2
3
4
Number of Photobleaching Steps
Intensity (a.u.)
0
400
800
0
50
100
150
Time (seconds)
D
Normalized Nucleosome Signal Intensity
0.5
0.6
0.7
0.8
0.9
1.0
0
20
40
60
80
100
Time (minutes)

Figure S6. Nucleosome-Bound DNA Reconstitution Using Xenopus Histones on λ-DNA, Related to Figure 7

(A) Coomassie staining and ATTO-647N scan of SDS-PAGE of ATTO-647N-labeled xenopus histone octamers.

(B) Distribution of numbers of reconstituted nucleosomes on DNA molecules (n = 103).

(C) Distribution of numbers of photobleaching steps at ATTO-647N signal puncta on DNA (n = 92). Inset: representative trace showing two–step photobleaching.

(D) Normalized nucleosome signal intensity over time, under single-molecule DNA curtain assay conditions. Shaded region indicates standard deviation across DNA molecules (n = 190).
